# Enhanced Multi-Strategy Improved Animated Oat Optimization Algorithm and Its Engineering Application

**DOI:** 10.3390/biomimetics11070486

**Published:** 2026-07-10

**Authors:** Sunde Wang, Beilei Yin, Pu Wang, Zihao Cheng

**Affiliations:** 1 School of Electronics and Electrical Engineering, Wenzhou University of Technology, Wenzhou 325035, China; 20250014@wzut.edu.cn; 2 Engineering Technology Department, Shanghai Caoyang Vocational School, Shanghai 200333, China; yinbeilei197905@163.com; 3 School of Computer Engineering, Hubei University of Arts and Science, Xiangyang 441053, China; 4 College of Control Science and Engineering, Zhejiang University, Hangzhou 310027, China; chengzihao@zju.edu.cn

**Keywords:** Animated Oat Optimization Algorithm, Sinusoidal chaotic map, disturbance factor, adaptive *t*-distribution, swarm intelligence optimization, engineering design optimization

## Abstract

To address the inherent limitations of the traditional Animated Oat Optimization Algorithm (AOO), including poor uniformity of initial random population distribution and insufficient dynamic balance between global exploration and local exploitation, this paper proposes an Enhanced Animated Oat Optimization Algorithm (EAOO) incorporating multi-strategy improvements. First, the Sinusoidal chaotic map is introduced to replace the original random initialization method. Leveraging the ergodicity and uniformity of chaotic sequences, the spatial distribution of the population is optimized, and the diversity of the initial population is significantly enhanced. Second, a nonlinear disturbance factor is embedded into the position update of leaders during both the exploration and exploitation phases, enabling dynamic and adaptive adjustment of the search range. This effectively balances the algorithm’s capabilities in global exploration and local exploitation. Finally, an adaptive *t*-distribution mutation operator, combined with a dynamic selection strategy, is integrated. The degrees of freedom are adaptively adjusted throughout the iterative process, allowing the algorithm to switch between global escape and local fine-search modes, thereby overcoming the premature convergence deficiency of the original algorithm. Simulation and comparative experiments are conducted based on the CEC2017 and CEC2020 benchmark function suites. Systematic evaluations are carried out from multiple perspectives, including optimization accuracy, convergence speed, and statistical significance. The experimental results demonstrate that the proposed EAOO achieves superior comprehensive performance across various complex function types—including unimodal, multimodal, hybrid, and composite functions—exhibiting higher optimization accuracy, faster convergence speed, and stronger robustness. Statistical tests further confirm the significant performance differences between EAOO and the compared algorithms. Furthermore, EAOO is applied to two typical constrained engineering optimization problems: welded beam design and pressure vessel design. The simulation results show that EAOO yields better structural design parameters and lower manufacturing costs, demonstrating outstanding practical value and broad application prospects in solving high-dimensional, nonlinear, constrained engineering optimization problems.

## 1. Introduction

With the rapid development of advanced industries including modern industrial manufacturing, aerospace equipment design, civil structural optimization, wireless sensor network deployment and new energy dispatch, engineering systems have grown drastically more complex and high-dimensional [1,2]. Traditional low-dimensional linear convex optimization approaches can hardly satisfy real industrial demands. Instead, optimization problems featuring high dimensionality, strong nonlinearity, multiple local extrema and rigid constraints have become prevalent [3]. Such tasks often contain non-differentiable objective functions with unavailable gradient information and dense local optima confined within narrow feasible domains, which renders gradient-driven deterministic solvers such as Newton’s method and gradient descent infeasible [4,5]. Conventional deterministic algorithms are built upon strong prerequisites that objective functions are smooth and convex. When tackling high-dimensional non-convex landscapes, they easily suffer from the curse of dimensionality, iterative stagnation and premature convergence, producing solutions whose accuracy and efficiency fail to meet industrial design criteria [6]. Under such circumstances, novel efficient optimization solvers are urgently required. Against this background, swarm intelligence (SI) optimizers have attracted extensive research attention by virtue of their gradient-free characteristic, powerful global search ability, flexible architecture and strong robustness [7,8,9], emerging as a dominant research branch for complex engineering optimization. Swarm intelligence algorithms mimic natural evolutionary behaviors of biological groups (e.g., foraging and migration) as well as plant-specific survival patterns such as seed dispersal and adaptive growth [10,11]. Via information sharing and collaborative iteration among individuals, these methods autonomously excavate high-quality feasible regions throughout the solution space, successfully mitigating the inherent flaw of traditional gradient-based methods that readily converge to local optima [12,13]. Decades of continuous development have enabled swarm intelligence techniques to be widely deployed in structural design, path planning, parameter identification and energy scheduling [14].

The No Free Lunch (NFL) theorem theoretically verifies that no single metaheuristic can achieve state-of-the-art performance over all optimization tasks [15]. Every swarm intelligence algorithm possesses its unique applicable domains and inherent performance bottlenecks. Animal-derived metaheuristics including Particle Swarm Optimization (PSO) [16] and Grey Wolf Optimizer (GWO) [17] have undergone mature development, yet they commonly suffer from uneven initial population distribution, sluggish late-stage convergence and severe performance deterioration under high-dimensional settings [18]. In contrast, plant-inspired intelligent algorithms emerged much later. Even so, plants have evolved distinctive survival strategies over hundreds of millions of years—long-distance seed dispersal and hygroscopic deformation included—that perfectly match the core logic of metaheuristic search, endowing this branch with profound theoretical and engineering research value [19,20].

The Animated Oat Optimization Algorithm (AOO), a newly proposed plant-based swarm intelligence optimizer dated to 2025 [21], draws inspiration from three seed propagation modes of animated oats: passive diffusion via natural carriers, self-propelled rolling driven by hygroscopic deformation, and energy-releasing ejection when colliding with barriers. Based on these bionic mechanisms, AOO establishes a three-stage iterative pipeline consisting of population initialization, global exploration and local exploitation [22,23]. Benefiting from its compact structure and a small set of hyperparameters, AOO exhibits promising performance on low-dimensional function optimization and wireless sensor node localization. Nevertheless, thorough investigations have uncovered multiple critical defects of the vanilla AOO. Its purely random initialization easily triggers individual clustering and incomplete coverage of the search domain, severely degrading initial population diversity [24]. The rigid fixed update rule lacks adaptive regulation, making it impossible to dynamically coordinate global exploration and local exploitation. Moreover, the absence of mutation-based perturbation and local optimum escape operators causes AOO to become trapped frequently when solving multimodal problems, with its optimization precision and stability declining sharply for high-dimensional constrained engineering tasks [25]. Existing studies on AOO remain preliminary, restricted to fundamental function tests and trivial application cases; no systematic framework has been proposed to improve its initialization, iterative update and escape mechanisms. Meanwhile, multi-strategy hybrid improvement frameworks have reached full maturity, as represented by enhanced wild horse optimizers [26,27]. Techniques such as chaotic initialization and adaptive perturbation can effectively offset the defects of primitive metaheuristics, offering solid references for upgrading AOO [28]. Accordingly, developing multi-strategy improved AOO variants, validating their optimization performance and extending their engineering applications carry great theoretical significance and practical value.

Aiming at the shortcomings of original AOO including poor initial population distribution, local optimum trapping, slow convergence and weak high-dimensional robustness, this paper proposes an Enhanced Animated Oat Optimization Algorithm (EAOO) referring to multi-strategy improvement paradigms. The main innovations are as follows:

(1) The Sinusoidal chaotic map is introduced to replace random initialization. Relying on the randomness and ergodicity of chaotic sequences, it optimizes the coverage of the initial population, eliminates aggregation and search blind spots, and improves initial diversity from the source.
(2) A nonlinear adaptive disturbance factor is designed and embedded into the leader update formula to dynamically adjust the search range, realizing global exploration in the early stage and local fine exploitation in the later stage.
(3) An adaptive *t*-distribution mutation operator combined with a dynamic selection strategy is integrated. The degree of freedom is determined by iteration times. It approximates the Cauchy distribution in the early stage to strengthen global escape and approaches the Gaussian distribution in the later stage to improve local accuracy. Dynamic probability controls mutation frequency to balance convergence efficiency and precision.

## 2. Animated Oat Optimization Algorithm

Animated oat is an annual herb widely distributed in temperate regions worldwide with strong adaptability and reproductive capacity. Its unique hygroscopic awn structure forms three propagation modes: passive diffusion, autonomous rolling and energy storage ejection, which constitute the bionic basis of AOO [29,30]. The original AOO divides the optimization process into population initialization, global exploration and local exploitation. It switches stages through random probability and updates individual positions via bionic kinematics to achieve global optimization [31].

### 2.1. Population Initialization

Set the optimization dimension as *Dim* and population size as *N*. Let *UB_j_* and *LB_j_* be the upper and lower bounds of the *j*-th dimension. The original AOO randomly generates the population matrix [32].

(1) $$X=\left[\begin{array}{ccccc} x_{1,1} & \cdots & x_{1,j} & \cdots & x_{1,Dim}\\ x_{2,1} & \cdots & x_{2,j} & \cdots & x_{2,Dim}\\ \vdots & \vdots & \vdots & \vdots & \vdots \\ x_{N,1} & \cdots & x_{N,j} & \cdots & x_{N,Dim} \end{array}\right]$$

The position of the *i*-th individual in the *j*-th dimension is generated by uniform random numbers:

(2) $$x_{i,j}=r\times \left(UB_{j}-LB_{j}\right)+LB_{j}$$

where, i=1,2,…,N, j=1,2,…,Dim, the parameter *r* is a uniformly random number within the interval [0, 1].

Four core parameters including equivalent mass, main awn length, rolling eccentricity and dynamic balance factor are defined to simulate the physical characteristics of oat seeds [33].

(3) $$\left\{\begin{array}{l} m=0.5\times \frac{r}{dim}\\ L=N\times \frac{r}{dim}\\ e=0.5\times \frac{r}{dim}\\ c=1-{\left(\frac{t}{T}\right)}^{3} \end{array}\right.$$

In the equation: parameter *m* represents the equivalent mass of the seed. *L* denotes the main simulation length. *e* is the rolling eccentricity coefficient of the seed. *c* stands for the dynamic balance factor of exploration and exploitation. *t* indicates the current iteration count. *T* signifies the maximum iteration limit of the algorithm [34,35].

### 2.2. Global Exploration Stage

The global exploration stage simulates the long-distance random diffusion of oat seeds driven by wind and water. A dimension weight matrix is constructed first.

(4) $$W=\frac{c}{\pi }\times \left(2\times r_{dim}-1\right)⊗UB$$

In the formula, the parameter *r_dim_* is a random matrix that matches the dimension of the problem. ⊗ is a matrix dot multiplication operation [35].

According to the individual number modular operation rule, update positions in three categories to achieve global multi-mode search:

(5) $$\left\{\begin{array}{ll} X_{t+1}(i)=\frac{1}{N}\times \sum_{i=1}^{N}X_{t}(i)+W, & mod(i,N/10)=0\\ X_{t+1}(i)=X_{best}+W, & mod(i,N/10)=1\\ X_{t+1}(i)=X_{t}(i)+W, & else \end{array}\right.\;$$

In the formula, $X_{t}(i)$ represents the position of the *i*-th individual in the *t*-th generation. $X_{best}$ is the global optimal individual position of the current population [36].

### 2.3. Local Exploitation Stage

The exploitation stage switches between obstacle-free hygroscopic rolling and obstacle energy ejection with a probability of 0.5, and Lévy flight is introduced to enhance local randomness and refinement.

1. Moisture absorbing rolling mode

(6) $$A=UB-\left|\frac{UB\times t\times sin(2\times \pi \times r)}{T}\right|$$

(7) $$R=\left(m\times e+L^{2}\right)\times \frac{r_{dim}(A,A)}{dim}$$

Levy flight step calculation formula:

(8) $$Levy(dim)=0.01\;\times \frac{\mu \times \sigma }{|v{|}^{1/\beta }}$$

(9) $$\sigma ={\left(\frac{\Gamma (1+\beta )\times sin\left(\frac{\pi \times \beta }{2}\right)}{\Gamma \left(\frac{1+\beta }{2}\right)\times \beta \times 2^{\left(\frac{\beta -1}{2}\right)}}\right)}^{1/\beta }$$

Individual location update:

(10) $$X_{t}(i)=X_{best}+R+c\times Levy(dim)⊗X_{best}$$

2. Launch mode when encountering obstacles

(11) $$B=UB-\left|\frac{UB\times t\times cos(2\times \pi \times r)}{T}\right|$$

Define ejection motion characteristic parameters:

(12) $$\left\{\begin{array}{l} k=0.5+0.5\;\times r\\ x=3\;\times \frac{r}{dim}\\ \theta =\pi \;\times r\\ \alpha =\frac{1}{\pi }\;\times e^{\frac{r^{\prime }}{T}} \end{array}\right.$$

Calculation of ejection displacement:

(13) $$J=\frac{2\;\times k\times x^{2}\;\times sin(2\theta )}{mg}\;\times \frac{r_{dim}(-B,B)}{dim}\;\times (1-\alpha )\;$$

(14) $$X_{t}(i)=X_{best}+J+c\times Levy(dim)⊗X_{best}$$

As a novel plant-inspired bionic intelligent optimization algorithm, the Animated Oat Optimization Algorithm (AOO) offers advantages such as a simple iterative structure, few control parameters, and a unique seed propagation mechanism [37]. Its principle is clear, and it exhibits stable convergence performance in low-dimensional scenarios, demonstrating good basic solving capability for simple optimization problems [38]. However, the original AOO suffers from several inherent deficiencies. The random initialization strategy leads to poor population distribution uniformity and insufficient spatial search coverage, which can easily result in blind areas during the early iteration stage. The fixed position update rules lack adaptive adjustment capability and fail to dynamically balance global exploration with local exploitation, making the algorithm prone to local optima and premature convergence in later iterations. Furthermore, the algorithm has weak anti-interference ability and poor adaptability to high-dimensional problems, with its optimization accuracy and iterative stability degrading significantly when solving complex optimization problems characterized by multi-peak distortion, strong coupling, and multiple constraints. These limitations restrict its application in complex engineering optimization scenarios. To address the aforementioned shortcomings of the original AOO, this paper proposes an enhanced multi-strategy Animated Oat Optimization Algorithm (EAOO). Through the collaborative optimization of multiple improved strategies, the performance defects of the original algorithm are systematically compensated, effectively enhancing the convergence speed, optimization accuracy, and engineering robustness of the algorithm.

## 3. Enhanced Animated Oat Optimization Algorithm

### 3.1. Sinusoidal Chaotic Map Initialization

Random initialization of original AOO easily causes uneven distribution and low population diversity. Chaotic maps possess randomness and ergodicity. Compared with Logistic and Circle maps, the Sinusoidal map has smoother sequence distribution and wider value range, which can generate uniformly distributed initial individuals and improve population diversity effectively [39].

The iterative formula of Sinusoidal map is:

(15) $$x_{k+1}=ax_{k}^{2}sin\left(\pi x_{k}\right)\;$$

where *a* = 2.3, *x_k_* denotes the *k*-th chaotic iteration value.

Replace the random number *r* in Formula (2) with the chaotic sequence generated by the Sinusoidal mapping, and reconstruct the initial population position [40]:

(16) $$x_{i,j}=x_{k}\;\times \left(UB_{j}-LB_{j}\right)+LB_{j}$$

This method enables the initial population to cover the whole search space uniformly and provides a good foundation for global exploration.

### 3.2. Nonlinear Disturbance Factor-Based Leader Adaptive Update

The leader position update of original AOO lacks disturbance and tends to stagnate in the later iteration. A nonlinear disturbance factor is introduced to adjust adaptively with iteration times [41]. It expands the search range far from the optimal solution and shrinks the radius near the optimum to balance exploration and exploitation. Calculation formula for disturbance factor:

(17) $$g=\left\{cos[{\left(1-iter/maxiter\right)}^{2}\;\times \pi ]\right\}+\alpha$$

In the formula, α = 4 is a fixed adjustment parameter; the disturbance factor varies nonlinearly with the number of iterations and has adaptive characteristics. Update formula for leader position in the development phase after improvement [42]:

(18) $$\left\{\begin{array}{ll} X_{t+1}(i)=\left[X_{best}+J+c\;\times Levy(dim)⊗X_{best}\right]\;\times \frac{1}{g}, & r>0.5\\ X_{t+1}(i)=\left[X_{best}+R+c\;\times Levy(dim)⊗X_{best}\right]\;\times \frac{1}{g}, & r\leq 0.5 \end{array}\right.\;$$

The disturbance factor can adaptively adjust the search step size, expanding the exploration range when moving away from the optimal area and reducing the search radius when approaching the optimal solution, effectively balancing global exploration and local development.

### 3.3. Adaptive t-Distribution and Dynamic Selection Strategy

The *t*-distribution has the characteristics of both Cauchy and Gaussian distributions. With small degrees of freedom in the early iteration, it approximates the Cauchy distribution for strong global disturbance [43]. With increasing degrees of freedom in the later stage, it approaches the Gaussian distribution for local precise searching. This paper constructs an adaptive *t*-distribution mutation operator taking iteration times as the degree of freedom.

(19) $${\bar{X}}_{i,t}=X_{i,t}+X_{i,t}\times t(iter)\;$$

In the formula, t(iter)  is a *t*-distributed random variable with iteration times as degrees of freedom. $X_{i,t}$ is the position of the mutated individual.

To avoid increasing computational complexity due to full staff variation, dynamic selection probability is introduced [44]:

(20) $$q=w_{1}-w_{2}\;\times \left(\;maxiter\;-\;iter\;\right)\;/\;maxiter\;$$

In the formula: $w_{1}$ = $w_{2}$ = 0.8; in the early stage of iteration, the *q* value is relatively high, and there is a high probability of performing *t*-distribution mutation to enhance global search. In the later stage, the *q* value decreases while retaining the original development mechanism, balancing convergence speed and accuracy.

### 3.4. Algorithm Implementation Steps and Pseudocode

Step 1: Initialize the basic parameters of the algorithm, set the population size *N*, maximum iteration times *T*, optimization dimension *Dim*, search space upper and lower bounds *UB*/*LB*, fix the Sinusoidal mapping, perturbation factor, and *t*-distribution related constants.

Step 2: Use Sinusoidal chaotic mapping to generate chaotic sequences, and initialize the population using Formula (16) instead of the original random generation method.

Step 3: Calculate the fitness values of all individuals, filter and record the global optimal individual position *X_best_* and optimal fitness *F_best_*.

Step 4: Enter the iteration loop, calculate the seed feature parameters according to Formula (3), calculate the adaptive disturbance factor according to Formula (17), and calculate the dynamic mutation probability according to Formula (20).

Step 5: Generate a 0.5 random probability, determine whether to enter the global exploration or local development stage, update individual positions according to corresponding formulas, and introduce disturbance factors to adaptively adjust the search range.

Step 6: Generate a random number and compare it with the dynamic probability *q*. If the conditions are met, perform adaptive *t*-distribution mutation and update individual positions;

Step 7: Perform boundary constraint correction on individuals who have crossed the boundary, recalculate fitness values, and update the global optimal individual.

Step 8: Determine whether the maximum number of iterations has been reached. If it has, terminate the iteration and output the optimal solution. Otherwise, continue the loop iteration.

The pseudocode of the EAOO algorithm is shown in Algorithm 1.
**Algorithm 1.** EAOO algorithm pseudocode.**Input:**      Population size *N*, maximum iteration *T*, dimension *Dim*, search boundary *UB*/*LB* **Output:**    Global optimal position *X_best_*, global optimal fitness *F_best_*
1:  Algorithm parameter initialization, fixed constant assignment: *a* = 2.3, *α* = 4, *w*_1_ = *w*_2_ = 0.8.
2:    Generate chaotic sequences using the Sinusoidal mapping Formula (15). 
3:    Initialize the individual positions of the population based on Formula (16) chaos. 
4:  Calculate the fitness of all individuals, initialize *X_best_* and *F_best_*.
5:  for t = 1 to *T*

6:  Calculate the basic parameters of *m*, *L*, *e*, and *c* according to Formula (3). 
7:  Calculate the nonlinear disturbance factor *g* according to Formula (17) 
8:  Calculate the dynamic mutation selection probability *q* according to Formula (20).
9:  for i = 1 to *N*

10:          if *rand* > 0.5 
11:                Perform global exploration and update individual positions according to Formulas (4) and (5).
12:          else 
13:                    Perform local development and update individual positions according to Formulas (6) and (18). 
14:          end if 
15:          if *rand* > *q*

16:                Perform adaptive *t*-distribution mutation operation according to Formula (19). 
17:          end if 
18:            Boundary constraint processing, correcting out of bounds individuals 
19:            Recalculate individual fitness 
20:          Update global optimum *X_best_* and *F_best_*

21:  end for 
22:  end for
23:    Output optimal position *X_best_* and optimal fitness *F_best_*.

### 3.5. Algorithm Complexity Analysis

Algorithm complexity serves as a critical evaluation indicator to assess the operational efficiency and resource consumption of intelligent optimization algorithms, and it fundamentally governs the algorithm’s simulation performance and engineering applicability. As commented by reviewers, pure theoretical complexity analysis is insufficient to comprehensively validate algorithmic efficiency; hence, this section first conducts rigorous theoretical analyses on the time and space complexity of the enhanced Animated Oat Optimization Algorithm (EAOO), and the corresponding computational efficiency experiments are supplemented in subsequent sections to further verify the effectiveness of the improved strategies. The primary analysis objective is to confirm whether the multi-strategy improvement mechanisms proposed in this work can boost the optimization performance without introducing excessive computational overhead. For unified quantitative analysis, key hyperparameters are defined as follows: *N* denotes the population size, *T* represents the maximum iteration number, *Dim* refers to the optimization dimension, and *O*(*COF*) indicates the time complexity of single individual fitness evaluation. In terms of time complexity, the complete execution workflow of the EAOO consists of five core modules: population initialization, iterative parameter updating, individual position renewal, adaptive mutation, and global optimal value iteration. Specifically, the sinusoidal chaotic initialization adopted in EAOO only rearranges the initial population distribution to enhance population diversity without additional complex iterative operations, and its time complexity is maintained at *O*(*N* × *Dim*), which is identical to that of the original AOO. The calculation of iterative auxiliary parameters, including disturbance coefficients and dynamic mutation probabilities, belongs to constant-time arithmetic operations and exerts no impact on the overall time complexity. Moreover, the individual exploration–exploitation position update and *t*-distribution mutation modules are implemented via conventional dimensional traversal for all population individuals, whose computational cost per iteration is controllable.

By synthesizing the computational overhead of all functional modules, the overall time complexity of the proposed EAOO is summarized as *O*(*T* × *N* × (*Dim* + *COF*)). Such a result is consistent with that of the original AOO and other prevalent swarm intelligence optimizers, including PSO, GWO, and SSA. The theoretical outcome demonstrates that the improved strategies integrated into EAOO are lightweight plug-and-play mechanisms, which will not bring extra time consumption or increase computational burden.

From the perspective of space complexity, this metric characterizes the memory resource occupancy during algorithm execution. The data cached by EAOO during operation mainly covers the population position matrix, global optimal individual information, iterative auxiliary variables and pre-generated chaotic sequences. The core memory overhead originates from the two-dimensional population matrix with a space cost of *O*(*N* × *Dim*). Other auxiliary parameters, such as disturbance factors, dynamic mutation probabilities and oat seed characteristic coefficients, are fixed constant variables that occupy negligible memory resources. Consequently, the overall space complexity of EAOO is calculated as *O*(*N* × *Dim* + *T*), which is equivalent to the space consumption of the original AOO algorithm.

Combining the above theoretical complexity analysis and subsequent experimental results, the multi-strategy improvement schemes of EAOO (chaotic initialization, adaptive disturbance and dynamic *t*-distribution mutation) can significantly enhance the algorithm in terms of optimization accuracy, convergence performance and anti-premature convergence capability. Meanwhile, the proposed EAOO fully inherits the merits of the original AOO, such as lightweight architecture, low resource consumption and high portability. Neither dimensionality disaster nor redundant computational operations are observed. The complementary theoretical analysis and experimental validation collectively prove that EAOO possesses outstanding operational efficiency and promising application potential for complex practical engineering optimization tasks.

## 4. Algorithm Comparison and Experimental Analysis

To scientifically, objectively and comprehensively validate the overall optimization capability of the proposed Enhanced Multi-strategy Improved Animated Oat Optimization Algorithm (EAOO), a series of comparative simulation experiments are designed in this chapter. Eight state-of-the-art swarm intelligence optimizers from domestic and international literature are chosen for benchmark comparison: the proposed EAOO, original AOO, Black-winged Kite Algorithm (BKA) [45], Sparrow Search Algorithm (SSA) [46], Harris Hawks Optimization (HHO) [47], Dung Beetle Optimizer (DBO) [48], Grey Wolf Optimizer (GWO) [49], and Hippopotamus Optimization Algorithm (HO) [50]. The comparative pool covers four major categories of metaheuristics: novel plant-inspired algorithms, mammal-based optimizers, bird swarm algorithms and insect intelligence methods. Such diversified comparison baselines enable a thorough horizontal validation of EAOO’s effectiveness and overall competitive performance.

The simulation framework consists of two experimental modules: standard benchmark function testing and constrained engineering optimization testing. The internationally recognized CEC2017 and CEC2020 complex benchmark suites are adopted for numerical verification, which contain unimodal, multimodal, hybrid, and rotated shifted composite functions. These diversified test functions jointly assess five core algorithm indicators: convergence speed, optimization precision, global local-optimum escape capacity, high-dimensional stability, and anti-interference performance. For practical validation, two classic constrained mechanical design problems—welded beam optimization and pressure vessel optimization—are selected to mimic real-world industrial optimization scenarios with complicated coupled constraints. Rather than presenting raw data tables, this work delivers in-depth textual analysis from multiple dimensions including iterative mechanism, convergence profiles, search logic, performance gaps and overall superiority, so as to clearly highlight EAOO’s comprehensive strengths against the other seven competitors.

All simulations are conducted under identical hardware and software setups to guarantee experimental fairness, consistency and reproducibility. MATLAB R2023b serves as the unified simulation platform to eliminate environmental discrepancies across the eight algorithms. To avoid biases induced by inconsistent parameter configurations, all algorithms share identical universal hyperparameters: population size *N* = 30, maximum iteration count *T* = 1000, search domain bound [−100, 100], and fixed optimization dimension of 30. Each algorithm runs 30 independent repeated trials to mitigate stochastic noise from random initialization, rendering experimental conclusions reliable and statistically meaningful. In addition, the native default parameter settings of each compared algorithm are preserved without manual fine-tuning, so their inherent search characteristics remain intact during comparison.

### 4.1. Analysis of CEC2017 Test Function Experimental

**Figure 1:**
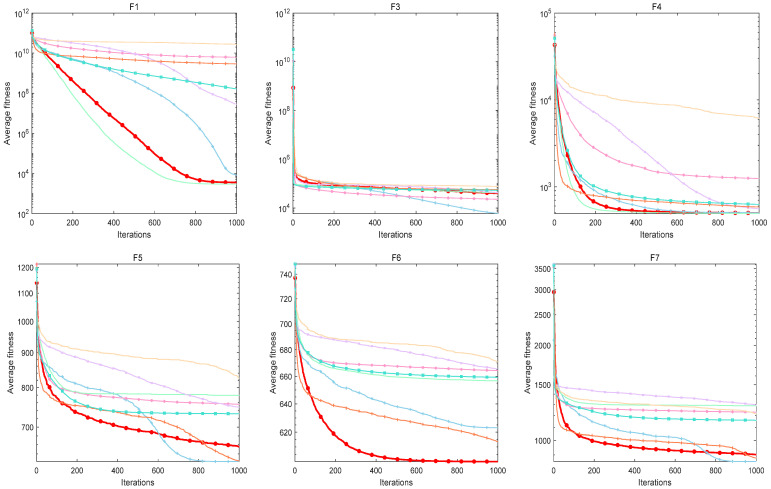

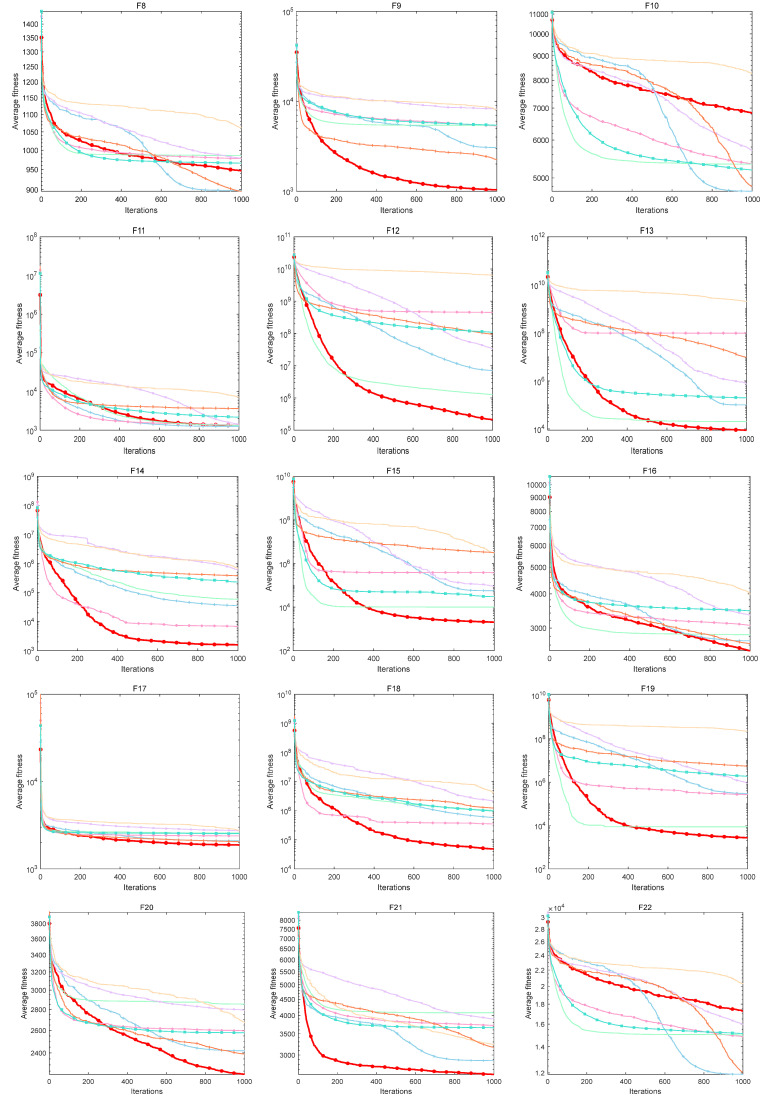

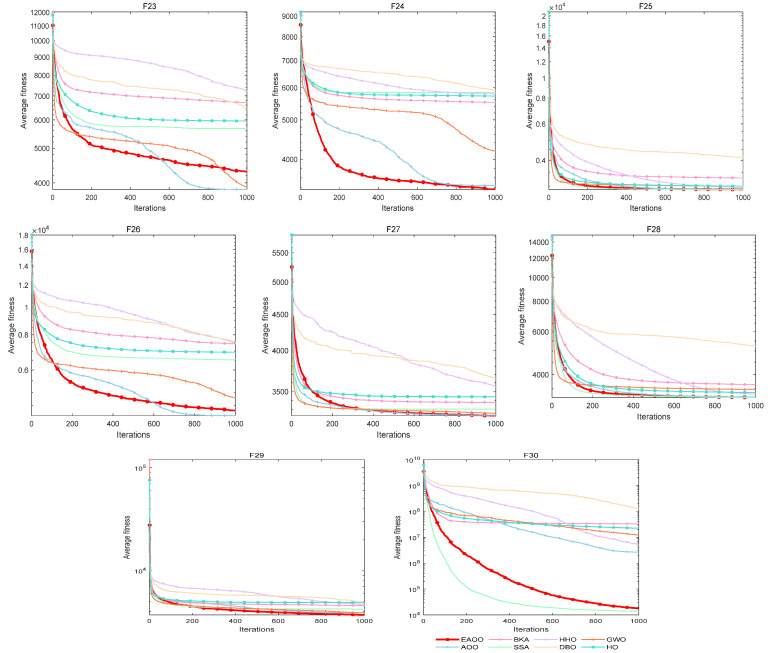
Comparison of average convergence curves of CEC2017.

**Figure 2:**
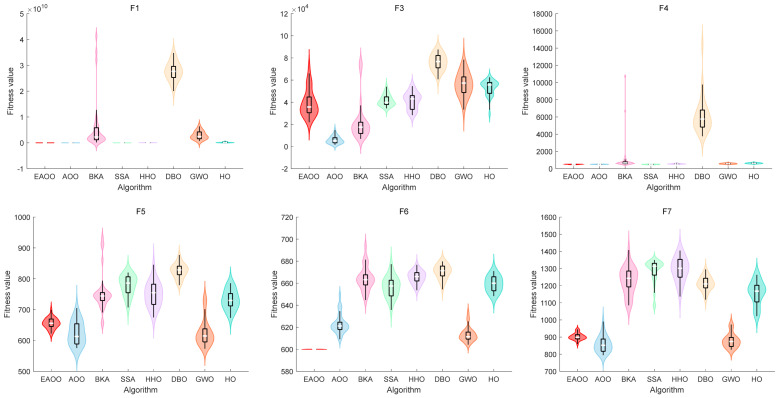

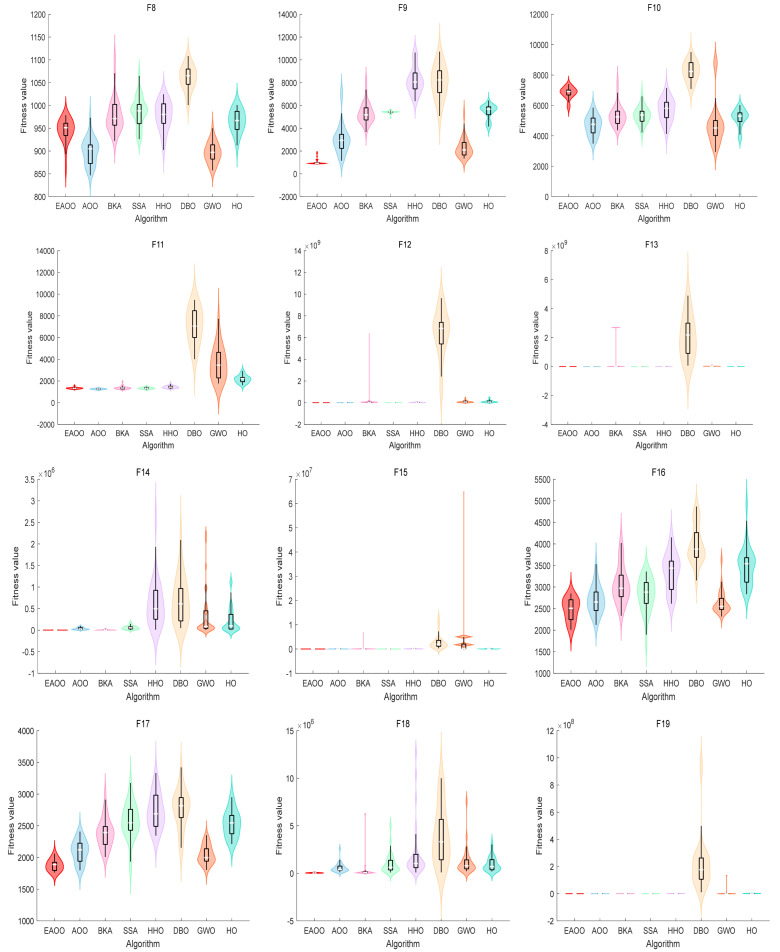

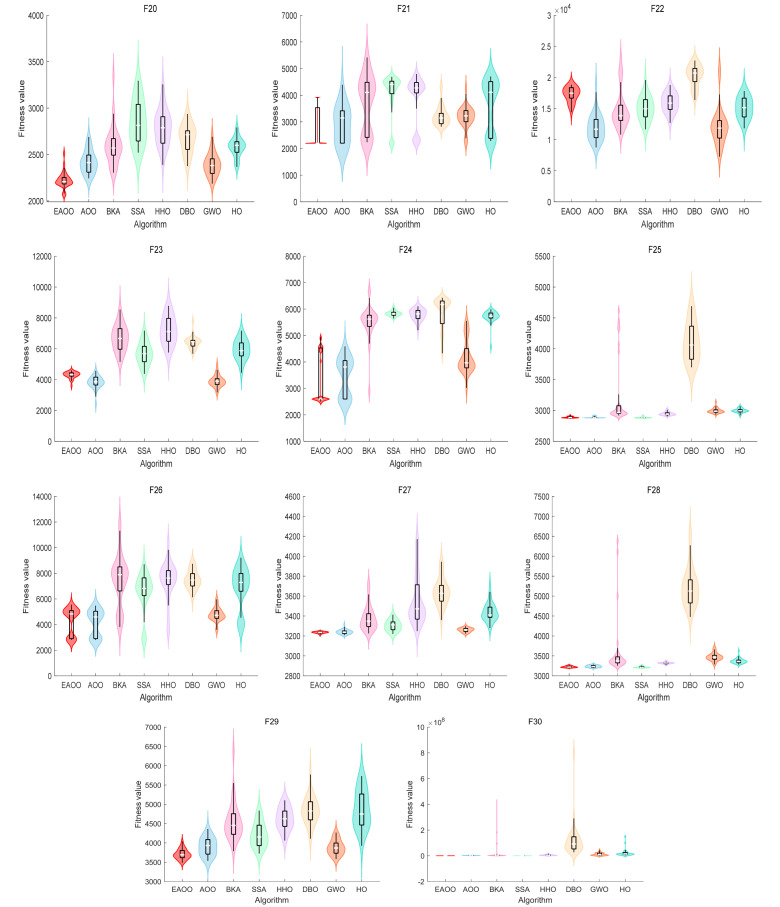
Comparison of violin plots of CEC2017.

The CEC2017 test function set is currently one of the most authoritative standard test sets in the field of intelligent optimization algorithms, consisting of 30 sets of complex test functions. The functions introduce complex mathematical transformations such as offset, rotation, nonlinear coupling, and multi extremum traps, which can comprehensively simulate complex nonlinear optimization problems. This article divides functions into four categories based on their characteristics: unimodal test functions, multimodal test functions, mixed functions, and combination functions. Detailed analysis is conducted from the perspectives of convergence speed, optimization accuracy, resistance to premature convergence, and iterative stability. The comparison of the average convergence curves of CEC2017 is shown in Figure 1. The violin plot comparison of CEC2017 algorithm data is shown in Figure 2. The data comparison of eight algorithms CEC2017 is shown in Table 1. The ranking matrices of eight algorithms CEC2017 are shown in Table 2. The comparison of Wilcoxon rank sum results for CEC2017 is shown in Table 3. The Win_Tie_Loss statistics for CEC2017 are shown in Table 4.

The experimental results indicate that, during the iteration process for unimodal functions, all eight algorithms are able to converge continuously toward the optimal position, yet the differences in convergence performance are pronounced. Among them, the proposed EAOO algorithm exhibits the steepest convergence trend. In the early iteration stage, it relies on Sinusoidal chaotic mapping to achieve a uniform population distribution and rapidly locks onto the optimal search region. In the middle stage, it adaptively reduces the search step size using a nonlinear disturbance factor, progressively approaching the global optimum. In the later stage, combined with Lévy flight for fine-grained search, the solution asymptotically approaches the theoretical optimum. In contrast, among the other seven comparison algorithms, the original AOO suffers from low search efficiency in the early stage due to random initialization and experiences convergence stagnation in the middle-to-late iterations. The BKA and GWO algorithms exhibit moderate convergence speed but weak optimization capability in later stages. The SSA possesses strong local search ability but insufficient global search, making it prone to premature convergence. The HHO, DBO, and HO algorithms show significant iteration fluctuations, with evident oscillations in their convergence curves even in unimodal scenarios, indicating insufficient stability. From the perspective of convergence mechanisms, the disturbance factor introduced in EAOO enables dynamic adjustment of the search range, addressing the fixed-step update limitation of the original AOO, thereby achieving faster convergence speed and higher solution accuracy in unimodal function optimization.

The optimization difficulty of multimodal functions is substantially higher than that of unimodal functions. Most traditional swarm intelligence algorithms tend to fall into local extrema and fail to escape. Among the eight algorithms compared in this study, SSA, GWO, and HO generally exhibit premature convergence on multimodal functions, becoming trapped in suboptimal local solutions by the middle iteration. Although BKA, HHO, and DBO possess a certain ability to escape local optima, the escape process is time-consuming, and their iteration curves exhibit severe fluctuations. Due to the lack of a mutation disturbance mechanism, the original AOO algorithm demonstrates weak escape capability when faced with dense extremum traps, resulting in limited optimization performance. In contrast, the EAOO algorithm perfectly addresses multimodal optimization challenges through its adaptive *t*-distribution mutation strategy. In the early iteration, the *t*-distribution has low degrees of freedom, approximating the characteristics of a Cauchy distribution, which significantly perturbs individual positions and endows the population with strong jumping ability to quickly escape local optima. In later iterations, as the degrees of freedom increase, the distribution approaches a Gaussian distribution, enabling small-scale fine disturbances to optimize the neighborhood around the optimum. Meanwhile, the dynamic selection probability strategy reasonably controls the mutation trigger frequency, preventing excessive mutation from disrupting high-quality individual structures. Comprehensive iteration performance demonstrates that EAOO possesses strong anti-interference ability and outstanding escape performance in multimodal complex scenarios, significantly outperforming the other seven comparison algorithms.

In hybrid and composite function tests, all seven comparison algorithms exhibit performance degradation to varying degrees. Among them, the GWO and HO algorithms are most affected by rotation transformation, suffering from severe deviation in search direction. The population diversity of SSA and DBO declines rapidly, leading to serious population homogenization in later stages. The BKA and HHO algorithms display significant oscillation in iterations, indicating poor stability. The original AOO algorithm has limited capacity to adjust the balance factor and fails to adapt to the distorted search space. The EAOO algorithm, by contrast, leverages the synergistic effects of multiple improvement strategies: chaotic initialization ensures uniform population distribution in complex spaces, the disturbance factor adapts to nonlinear distorted spaces, and adaptive mutation maintains population diversity. In highly coupled and strongly transformed complex functions, EAOO consistently maintains a stable convergence trend without noticeable performance degradation, and its comprehensive optimization capability far exceeds that of the comparison algorithms.

From the variation patterns of all CEC2017 convergence curves, it is evident that EAOO exhibits three-stage optimization characteristics: rapid exploration in the early stage, stable convergence in the middle stage, and fine-grained mining in the later stage. Among the eight algorithms, only EAOO shows no significant oscillation, premature stagnation, or late-stage divergence throughout the entire process, achieving the highest iteration fluency. Furthermore, the fluctuations across 30 repeated runs are very small, demonstrating that the algorithm has weak random interference and strong robustness. A comprehensive analysis shows that the multi-strategy improvement mechanism effectively compensates for the defects of the original AOO algorithm, endowing EAOO with overwhelming performance advantages across all types of functions.

### 4.2. Analysis of CEC2020 Test Function Experimental

**Figure 3:**
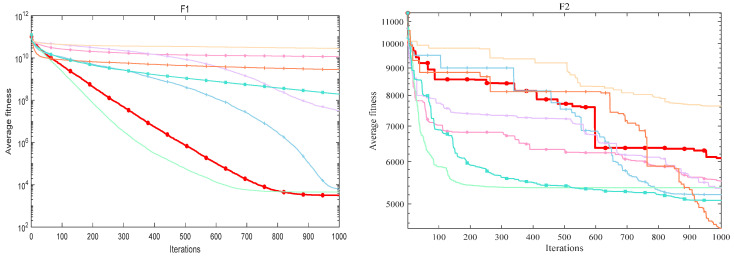

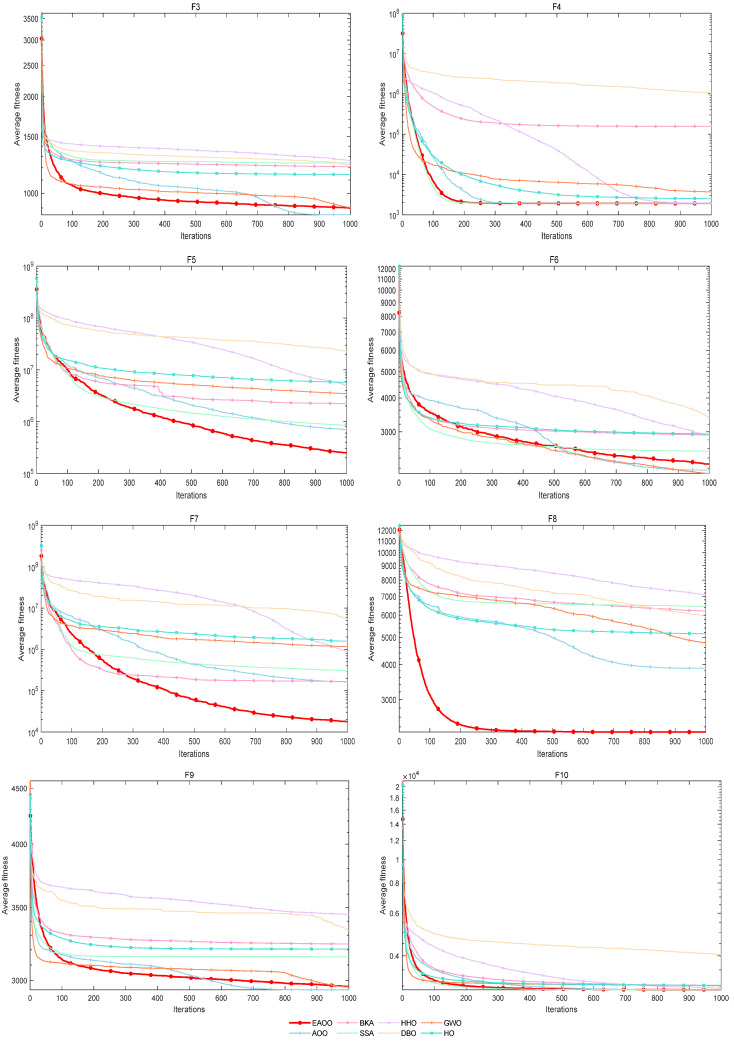
Comparison of average convergence curves for CEC2020.

**Figure 4:**
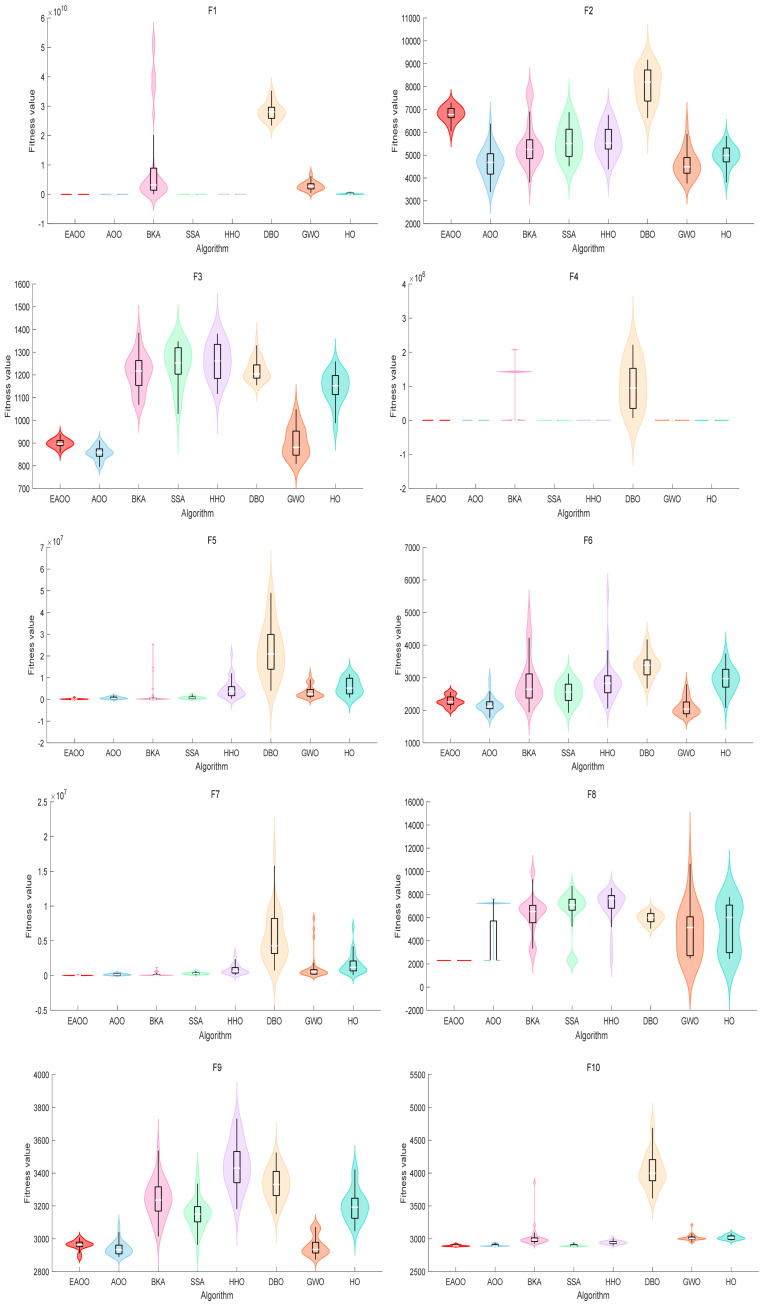
Comparison of violin plots of CEC2020 algorithm data.

The optimization difficulty of the CEC2020 test function set is higher than that of CEC2017. All test functions use strong offset, strong rotation, and highly nonlinear mathematical transformations, with high degree of surface distortion, narrow feasible range, and chaotic extreme value distribution. It is currently the authoritative test set for testing the engineering adaptability of algorithms. CEC2020 is closer to high-dimensional, strongly coupled, non-convex complex problems in real industrial optimization, which can further verify the practical optimization ability of the EAOO algorithm. The comparison of the average convergence curves of CEC2020 is shown in Figure 3. The violin plot comparison of CEC2020 algorithm data is shown in Figure 4. The data comparison of eight algorithms CEC2020 is shown in Table 5. The ranking matrices of eight algorithms CEC2020 are shown in Table 6. The comparison of Wilcoxon rank sum results for CEC2020 is shown in Table 7. The Win_Tie_Loss statistics of CEC2020 are shown in Table 8.

Under the test of CEC2020 complex transformation function, the accuracy gap of the eight algorithms is further widened. The seven comparison algorithms are seriously affected by spatial distortion, and the optimal solution is far away from the theoretical optimal. The original AOO algorithm is easy to fall into the narrow inferior feasible region due to uneven initial distribution, with low optimization accuracy. BKA and HHO algorithms have strong randomness and large discrete results of multiple operations. The search efficiency of SSA, DBO, GWO and HO algorithms decreases significantly in high-dimensional distorted space. In contrast, the EAOO algorithm realizes global uniform sampling by virtue of Sinusoidal chaotic mapping, accurately locates the high-quality region in the narrow feasible region, and cooperates with the disturbance factor to adaptively adjust the search step. It still maintains extremely high solution accuracy under strong interference environment, and the solution results of all test functions are better than the other seven comparison algorithms.

In the CEC2020 function iteration process, the convergence speed of EAOO always ranks first. The decline range in the early and middle iterations is significantly higher than that of other algorithms, and it can converge to high-quality optimal solutions with fewer iteration steps. The other seven algorithms generally have adverse phenomena such as slow convergence, curve jitter and local stagnation in the middle iteration. From the perspective of stability, EAOO has the lowest data dispersion and few outliers in multiple repeated experiments. In contrast, BKA, HHO and HO algorithms have strong randomness and large data fluctuation. SSA, DBO and GWO algorithms are easy to converge to local optimum fixedly with insufficient robustness. Stability analysis proves that the improved strategies of EAOO have strong coordination and the algorithm iteration mechanism is mature and reliable. With the continuous improvement of optimization dimension, most swarm intelligence algorithms will suffer from dimensional disaster, and the optimization performance is greatly attenuated. The high-dimensional simulation results in this paper show that the convergence accuracy and iteration speed of the other seven comparison algorithms decrease significantly after the dimension increases. Benefiting from the triple optimization mechanisms of chaotic initialization, adaptive disturbance and dynamic mutation, the EAOO algorithm still maintains a stable convergence trend in high-dimensional environment without obvious performance degradation. This characteristic proves that EAOO has the potential to deal with ultra-high-dimensional complex engineering problems and is more suitable for large-scale optimization scenarios in modern industry.

### 4.3. Engineering Optimization Application Analysis

To further verify the engineering solution ability of the EAOO algorithm under real industrial constraints, this paper selects two classic mechanical engineering optimization cases: welded beam design optimization and pressure vessel design optimization. Both types of engineering problems have the characteristics of multi-variable, multi-constraint, nonlinearity and cost minimization. The feasible region is narrow, the variable coupling is strong, and the local optimal solutions are dense. They are standard test models to verify the engineering practicability of intelligent algorithms. This chapter does not display experimental data tables, and focuses on analyzing the superiority of the EAOO algorithm from the perspectives of optimization mechanism, constraint adaptability and engineering optimization effect.

#### 4.3.1. Welded Beam Design Optimization

Welded beam design optimization takes the minimization of manufacturing cost as the optimization objective, including four optimization variables: weld thickness, weld length, beam height and beam width. The constraints cover multiple industrial hard indicators such as shear stress, bending stress, beam end deflection and structural size boundary. This engineering problem has strict constraints and a narrow feasible region. Traditional optimization algorithms are easy to converge to local inferior solutions and difficult to obtain the economically optimal scheme.

The welded beam design model adopted in this study is derived from the engineering optimization test problems proposed by Kumar et al. The problem consists of four continuous design variables and five inequality constraints, and its mathematical model can be formulated as follows:

(1) Design variables:

(21) $$x=\left[x_{1},x_{2},x_{3},x_{4}\right]=[h,l,t,b]$$

where h,l,t, and b denote the weld thickness, weld length, beam height, and beam thickness, respectively.

(2) Objective function:

(22) $$\min f(x)=0.04811tb(l+14)+1.10471h^{2}l$$

which represents minimizing the total fabrication cost of the welded beam subject to all constraints.

(3) Constraints:

(23) $$\left\{\begin{array}{l} g_{1}(x):h-b\leq 0,\\ g_{2}(x):\delta (x)-\delta_{\max}\leq 0,\\ g_{3}(x):P\leq P_{c}(x),\\ g_{4}(x):\tau (x)-\tau_{\max}\leq 0,\\ g_{5}(x):\sigma (x)-\sigma_{\max}\leq 0. \end{array}\right.$$

which correspond to the weld-to-beam thickness relationship constraint, maximum deflection constraint, buckling load constraint, maximum shear stress constraint, and maximum normal stress constraint, respectively.

(4) Variable bounds:

(24) $$\begin{array}{c} 0.125\leq h\leq 2\\ 0.1\leq l\leq 10\\ 0.1\leq t\leq 10\\ 0.1\leq b\leq 2 \end{array}$$

**Figure 5:**
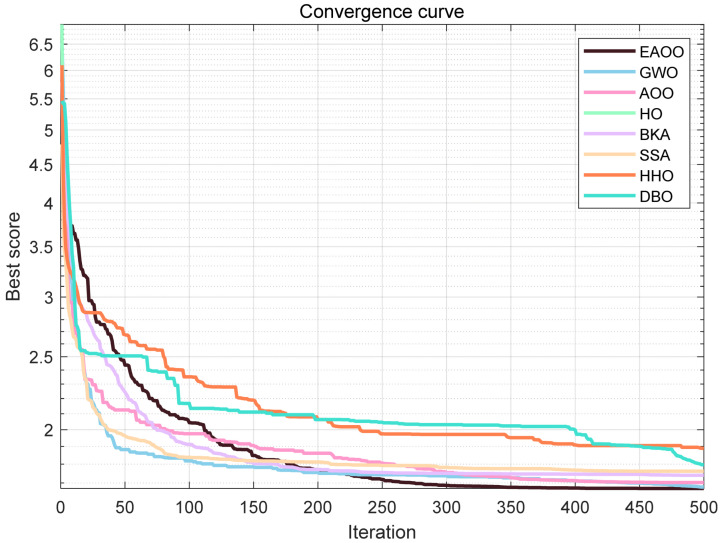
Comparison of average convergence curves for optimized welding beam design.

The comparison of average convergence curves for welding beam design optimization is shown in Figure 5. The comparison data of optimized welding beam design are shown in Table 9.

A comparison of the application results obtained from the eight algorithms indicates that the EAOO algorithm performs best in the welded beam optimization problem. By leveraging chaotic initialization, EAOO accurately locks onto the narrow feasible region, effectively preventing individuals from approaching the constraint boundaries. The nonlinear disturbance factor adaptively adjusts the search range of the four structural parameters, enabling coordinated matching optimization among them. Meanwhile, the adaptive *t*-distribution mutation helps escape local optimal parameter combinations and continuously iterates to refine a high-quality structural scheme. The resulting optimized welded beam exhibits a well-balanced structural parameter ratio, with all stress and deflection constraints satisfying industrial safety standards. Consequently, manufacturing cost is minimized without compromising structural strength or service safety.

In contrast, the other seven comparison algorithms exhibit notable deficiencies. The original AOO algorithm suffers from strong blindness in parameter search, leading to considerable structural redundancy and high cost. The BKA and HHO algorithms show large parameter fluctuations in later iterations, resulting in poor solution stability. The SSA and DBO algorithms tend to fall into local parameter combinations, lacking sufficient optimization depth. The GWO and HO algorithms demonstrate weak constraint-handling capability, with some optimized solutions approaching critical constraint boundaries and thus offering inadequate engineering safety margins. A comprehensive comparison reveals that EAOO possesses strong constraint-handling ability, high parameter optimization accuracy, and significantly better engineering adaptability than the other algorithms.

#### 4.3.2. Pressure Vessel Design Optimization

Pressure vessels are widely used in chemical, energy and aerospace fields. Their design optimization aims at minimizing tank production cost. The optimization variables include shell thickness, head thickness, vessel inner radius and cylindrical section length. The constraints of this model include material strength limit, pressure safety threshold and processing technology size limit. The variables have strong coupling correlation, and parameter changes restrict each other, with high optimization difficulty.

The goal of pressure vessel design is to minimize the total cost $f\left(x\right)$ while meeting the production needs. This problem includes four design variables: shell thickness $T_{s}$ (corresponding to design variable $x_{3}$) and head thickness $T_{h}$ (corresponding to design variable $x_{4}$), which are both integer multiples of 0.0625, and inner radius *R* (corresponding to design variable $x_{1}$) and vessel length *L* (corresponding to design variable $x_{2}$, excluding the head), both of which are continuous variables.

Objective function:

(25) $$\min f(x)=0.622,4x_{1}x_{3}x_{4}+1.778,1x_{2}x_{3}^{2}+3.166,1x_{1}^{2}x_{4}+19.84x_{1}^{2}x_{3}$$

(26) $$g_{1}(x)=-x_{1}+0.019,3x_{3}⩽0$$

(27) $$g_{2}(x)=-x_{2}+0.009,54x_{3}⩽0$$

Constraint conditions:

(28) $$g_{3}(x)=-\pi x_{3}^{2}x_{4}-\frac{4}{3}\pi x_{3}^{3}+1,296,000⩽0$$

(29) $$g_{4}(x)=x_{4}-240⩽0$$

Boundary constraints: $0⩽x_{1}⩽99,0⩽x_{2}⩽99,10⩽x_{3}⩽200,10⩽x_{4}⩽200$.

**Figure 6:**
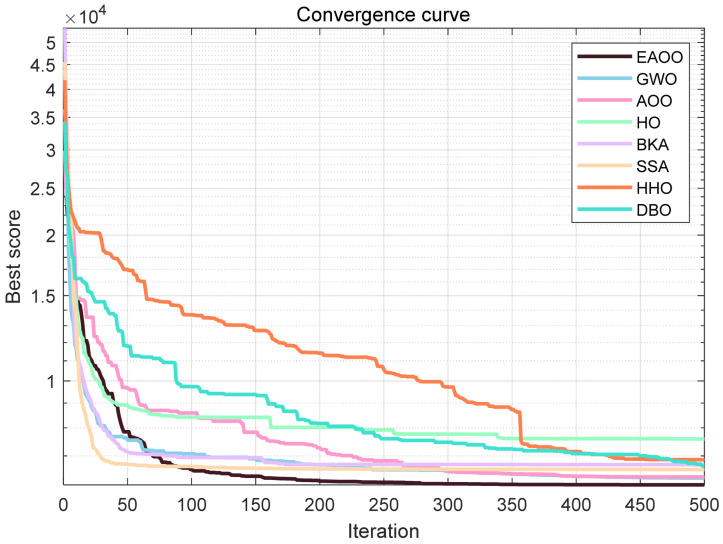
Comparison of average convergence curves for pressure vessel design optimization.

The comparison of average convergence curves for pressure vessel design optimization is shown in Figure 6. The comparative data of pressure vessel design optimization is shown in Table 10.

The optimization results of the eight algorithms indicate that the overall performance of the pressure vessel optimized by the EAOO algorithm is the best. During the iteration process, EAOO effectively balances the coupling relationships among thickness, radius, and length. While satisfying the constraints of pressure strength and processing requirements, it minimizes raw material consumption to the greatest extent, achieving a notable lightweight effect for the tank. Compared with the other algorithms, the vessel wall thickness optimized by EAOO is more reasonable, offering a moderate safety margin without redundant material waste, and successfully balancing safety, economy, and manufacturability. In contrast, the optimized schemes produced by the other seven algorithms exhibit various defects. The AOO and GWO algorithms result in excessively large design wall thickness and significant raw material waste. The BKA and HO algorithms yield unreasonable radius-to-length ratios, leading to low space utilization. Some iterative solutions of the SSA, HHO, and DBO algorithms approach the constraint limits too closely, resulting in poor engineering practicality and high potential safety risks. These comparisons demonstrate that the multi-strategy improvement mechanism of EAOO can effectively handle complex engineering constraints, possesses excellent collaborative optimization capability for coupled variables, and holds prominent industrial application value.

Combined with the analysis of the two engineering optimization cases, compared with the seven benchmark algorithms (AOO, BKA, SSA, HHO, DBO, GWO, and HO), the proposed EAOO algorithm exhibits stronger constraint adaptability, superior variable coupling processing ability, and enhanced global engineering optimization capability. Chaotic initialization improves the search ability within the feasible region, the disturbance factor balances the parameter search range, and adaptive mutation enhances the escape ability under complex operating conditions. Overall, EAOO can provide safe, economical, and reasonable structural design schemes under stringent industrial constraints, demonstrating good engineering implementability and industrial promotion value.

## 5. Conclusions

To tackle a set of inherent drawbacks of the original Animated Oat Optimization Algorithm (AOO)—namely uneven initial population distribution, a high tendency to fall into local optima in late iterations, inadequate trade-off between global exploration and local exploitation, low optimization precision, and poor robustness when handling high-dimensional complex problems—this paper develops a multi-strategy fusion improvement scheme and proposes an Enhanced Multi-strategy Animated Oat Optimization Algorithm (EAOO). First, sinusoidal chaotic mapping replaces the native random initialization scheme. This approach homogenizes the spatial distribution of the initial population, boosts initial population diversity, and fundamentally eliminates early-stage search blind zones and individual clustering issues. Second, a nonlinear adaptive disturbance factor is embedded to remodel the individual position updating rule, allowing search step sizes to self-adjust dynamically across the entire iterative cycle. This modification achieves a favorable balance between global exploration and local exploitation, addressing the original AOO’s rigid update rules and slow convergence rate. Building on the above two strategies, an adaptive *t*-distribution mutation operator is integrated, where the iteration count acts as the distribution’s degrees of freedom. Coupled with a dynamic selection probability strategy that governs mutation activation frequency, the algorithm delivers powerful global jumping capability in early iterations and precise local fine-tuning in later stages, which greatly strengthens its ability to escape local optima and elevates overall convergence accuracy. To fully validate the overall performance of the proposed EAOO, eight state-of-the-art swarm intelligence optimizers (EAOO, AOO, BKA, SSA, HHO, DBO, GWO, and HO) are selected for comprehensive comparative simulations using the internationally recognized CEC2017 and CEC2020 benchmark function suites. Experimental outcomes reveal that EAOO substantially outperforms the other seven competitors in terms of optimization accuracy, convergence speed, iterative stability, high-dimensional robustness, and anti-premature-convergence capability. Subsequently, EAOO is deployed to two classical constrained engineering optimization benchmarks: welded beam design and pressure vessel design. The experimental results further validate the superiority of EAOO for complex engineering tasks featuring multiple constraints, nonlinearity, and strongly coupled variables. EAOO yields superior structural design variables while cutting manufacturing costs, verifying its promising practical engineering value. Computational complexity analysis demonstrates that the integrated multi-strategy improvements introduce no extra time or space overhead; EAOO preserves the original AOO’s merits of concise architecture, lightweight computation, and high computational efficiency. In summary, the proposed EAOO effectively remedies most performance defects of the vanilla AOO. Nevertheless, its performance can be further refined for dynamic complex environments, multi-objective optimization tasks, and real-world engineering deployment.

Three promising directions are outlined for future research: algorithmic mechanism refinement, applicable scenario expansion, and extended engineering implementations. First, the current EAOO is only suitable for static single-objective optimization. Future work can introduce a multi-objective filtering mechanism and Pareto solution set maintenance strategy to construct a multi-objective EAOO variant, which can satisfy industrial multi-index collaborative optimization requirements that jointly minimize cost and weight while maximizing energy efficiency and structural safety. Second, the core iterative framework can be further upgraded by incorporating adaptive parameter adjustment, opposition-based learning, and hybrid mutation operators, so as to strengthen the algorithm’s adaptability to ultra-high-dimensional, severely disturbed, and time-varying dynamic optimization problems. Third, the application boundary of EAOO can be broadened to emerging intelligent engineering domains, including UAV path planning, neural network hyperparameter tuning, new energy load dispatching, and wireless sensor network node deployment. Such extensions can fully exploit the practical potential of plant-inspired metaheuristics and supply efficient, reliable alternative solutions for a wide range of intricate industrial optimization challenges.

## Figures and Tables

**Table 1:** Results of different swarm intelligence algorithms of CEC 2017.

Function	Metric	EAOO	AOO	BKA	SSA	HHO	DBO	GWO	HO
F1	Best	1.32 × 10^2^	2.33 × 10^3^	1.57 × 10^8^	1.12 × 10^2^	1.39 × 10^7^	2.01 × 10^10^	6.90 × 10^8^	4.22 × 10^6^
F1	Std	4.04 × 10^3^	5.23 × 10^3^	1.05 × 10^10^	2.76 × 10^3^	7.51 × 10^6^	3.58 × 10^9^	1.56 × 10^9^	1.62 × 10^8^
F1	Avg	3.54 × 10^3^	8.16 × 10^3^	6.27 × 10^9^	2.91 × 10^3^	2.82 × 10^7^	2.77 × 10^10^	2.93 × 10^9^	1.64 × 10^8^
F1	Time	3.14 × 10^−2^	6.09 × 10^−2^	2.15 × 10^−2^	3.01 × 10^−2^	3.19 × 10^−2^	2.77 × 10^−2^	2.66 × 10^−2^	3.26 × 10^−1^
F3	Best	2.19 × 10^4^	1.57 × 10^3^	6.90 × 10^3^	3.45 × 10^4^	2.83 × 10^4^	6.07 × 10^4^	3.33 × 10^4^	2.94 × 10^4^
F3	Std	1.23 × 10^4^	3.30 × 10^3^	1.85 × 10^4^	4.91 × 10^3^	7.63 × 10^3^	7.10 × 10^3^	1.03 × 10^4^	6.85 × 10^3^
F3	Avg	3.93 × 10^4^	5.91 × 10^3^	2.27 × 10^4^	4.15 × 10^4^	4.14 × 10^4^	7.63 × 10^4^	5.64 × 10^4^	5.32 × 10^4^
F3	Time	1.45 × 10^−2^	3.38 × 10^−2^	2.00 × 10^−2^	1.90 × 10^−2^	2.50 × 10^−2^	1.63 × 10^−2^	2.45 × 10^−2^	3.07 × 10^−1^
F4	Best	4.66 × 10^2^	4.70 × 10^2^	4.99 × 10^2^	4.53 × 10^2^	4.91 × 10^2^	3.76 × 10^3^	5.00 × 10^2^	5.30 × 10^2^
F4	Std	1.93 × 10^1^	1.61 × 10^1^	2.10 × 10^3^	2.13 × 10^1^	4.00 × 10^1^	2.13 × 10^3^	5.52 × 10^1^	5.31 × 10^1^
F4	Avg	4.97 × 10^2^	5.02 × 10^2^	1.24 × 10^3^	4.91 × 10^2^	5.54 × 10^2^	6.13 × 10^3^	5.85 × 10^2^	6.26 × 10^2^
F4	Time	1.35 × 10^−2^	3.34 × 10^−2^	1.60 × 10^−2^	1.71 × 10^−2^	2.77 × 10^−2^	2.10 × 10^−2^	3.18 × 10^−2^	3.58 × 10^−1^
F5	Best	6.22 × 10^2^	5.76 × 10^2^	6.54 × 10^2^	7.07 × 10^2^	6.75 × 10^2^	7.79 × 10^2^	5.73 × 10^2^	6.73 × 10^2^
F5	Std	1.69 × 10^1^	3.65 × 10^1^	6.53 × 10^1^	3.14 × 10^1^	3.90 × 10^1^	2.36 × 10^1^	4.44 × 10^1^	3.17 × 10^1^
F5	Avg	6.55 × 10^2^	6.24 × 10^2^	7.56 × 10^2^	7.79 × 10^2^	7.49 × 10^2^	8.26 × 10^2^	6.25 × 10^2^	7.33 × 10^2^
F5	Time	2.09 × 10^−2^	4.82 × 10^−2^	2.90 × 10^−2^	2.93 × 10^−2^	3.67 × 10^−2^	2.27 × 10^−2^	3.26 × 10^−2^	3.62 × 10^−1^
F6	Best	6.00 × 10^2^	6.09 × 10^2^	6.44 × 10^2^	6.36 × 10^2^	6.54 × 10^2^	6.54 × 10^2^	6.04 × 10^2^	6.48 × 10^2^
F6	Std	5.89 × 10^−2^	9.10 × 10^0^	1.11 × 10^1^	9.23 × 10^0^	5.68 × 10^0^	6.44 × 10^0^	6.72 × 10^0^	6.71 × 10^0^
F6	Avg	6.00 × 10^2^	6.23 × 10^2^	6.64 × 10^2^	6.57 × 10^2^	6.65 × 10^2^	6.71 × 10^2^	6.13 × 10^2^	6.59 × 10^2^
F6	Time	2.86 × 10^−2^	4.72 × 10^−2^	3.76 × 10^−2^	3.16 × 10^−2^	5.43 × 10^−2^	3.00 × 10^−2^	3.58 × 10^−2^	3.35 × 10^−1^
F7	Best	8.59 × 10^2^	7.95 × 10^2^	1.09 × 10^3^	1.08 × 10^3^	1.14 × 10^3^	1.12 × 10^3^	8.27 × 10^2^	1.02 × 10^3^
F7	Std	1.96 × 10^1^	4.61 × 10^1^	7.94 × 10^1^	7.17 × 10^1^	6.73 × 10^1^	4.04 × 10^1^	4.19 × 10^1^	7.01 × 10^1^
F7	Avg	9.03 × 10^2^	8.57 × 10^2^	1.23 × 10^3^	1.29 × 10^3^	1.30 × 10^3^	1.21 × 10^3^	8.80 × 10^2^	1.16 × 10^3^
F7	Time	1.65 × 10^−2^	3.66 × 10^−2^	2.12 × 10^−2^	1.97 × 10^−2^	3.49 × 10^−2^	2.68 × 10^−2^	3.69 × 10^−2^	3.69 × 10^−1^
F8	Best	8.62 × 10^2^	8.47 × 10^2^	9.22 × 10^2^	9.26 × 10^2^	9.02 × 10^2^	1.00 × 10^3^	8.57 × 10^2^	9.12 × 10^2^
F8	Std	2.50 × 10^1^	2.83 × 10^1^	3.87 × 10^1^	3.29 × 10^1^	3.11 × 10^1^	2.67 × 10^1^	2.24 × 10^1^	2.37 × 10^1^
F8	Avg	9.46 × 10^2^	8.99 × 10^2^	9.78 × 10^2^	9.86 × 10^2^	9.79 × 10^2^	1.06 × 10^3^	8.96 × 10^2^	9.66 × 10^2^
F8	Time	2.07 × 10^−2^	4.99 × 10^−2^	2.53 × 10^−2^	2.72 × 10^−2^	3.72 × 10^−2^	2.55 × 10^−2^	3.30 × 10^−2^	3.62 × 10^−1^
F9	Best	9.00 × 10^2^	1.15 × 10^3^	3.67 × 10^3^	4.94 × 10^3^	6.38 × 10^3^	5.07 × 10^3^	1.34 × 10^3^	4.02 × 10^3^
F9	Std	2.58 × 10^2^	1.38 × 10^3^	9.36 × 10^2^	1.23 × 10^2^	1.17 × 10^3^	1.43 × 10^3^	8.07 × 10^2^	6.68 × 10^2^
F9	Avg	1.04 × 10^3^	3.06 × 10^3^	5.33 × 10^3^	5.40 × 10^3^	8.22 × 10^3^	8.09 × 10^3^	2.25 × 10^3^	5.43 × 10^3^
F9	Time	3.17 × 10^−2^	5.67 × 10^−2^	3.43 × 10^−2^	2.70 × 10^−2^	3.92 × 10^−2^	2.16 × 10^−2^	3.15 × 10^−2^	3.41 × 10^−1^
F10	Best	5.81 × 10^3^	3.49 × 10^3^	4.37 × 10^3^	4.22 × 10^3^	4.12 × 10^3^	7.08 × 10^3^	2.95 × 10^3^	3.87 × 10^3^
F10	Std	3.87 × 10^2^	6.41 × 10^2^	6.96 × 10^2^	5.31 × 10^2^	7.33 × 10^2^	6.34 × 10^2^	1.50 × 10^3^	5.84 × 10^2^
F10	Avg	6.83 × 10^3^	4.69 × 10^3^	5.34 × 10^3^	5.34 × 10^3^	5.71 × 10^3^	8.26 × 10^3^	4.81 × 10^3^	5.20 × 10^3^
F10	Time	2.06 × 10^−2^	4.14 × 10^−2^	3.00 × 10^−2^	2.42 × 10^−2^	3.49 × 10^−2^	2.34 × 10^−2^	3.00 × 10^−2^	3.63 × 10^−1^
F11	Best	1.23 × 10^3^	1.18 × 10^3^	1.21 × 10^3^	1.21 × 10^3^	1.26 × 10^3^	4.01 × 10^3^	1.77 × 10^3^	1.61 × 10^3^
F11	Std	7.07 × 10^1^	4.76 × 10^1^	1.46 × 10^2^	7.58 × 10^1^	1.18 × 10^2^	1.57 × 10^3^	1.50 × 10^3^	3.49 × 10^2^
F11	Avg	1.33 × 10^3^	1.26 × 10^3^	1.37 × 10^3^	1.34 × 10^3^	1.45 × 10^3^	7.12 × 10^3^	3.64 × 10^3^	2.17 × 10^3^
F11	Time	2.16 × 10^−2^	4.78 × 10^−2^	2.67 × 10^−2^	2.55 × 10^−2^	3.59 × 10^−2^	2.77 × 10^−2^	3.19 × 10^−2^	3.61 × 10^−1^
F12	Best	2.94 × 10^4^	2.66 × 10^5^	1.95 × 10^6^	1.00 × 10^5^	5.84 × 10^6^	9.80 × 10^8^	5.68 × 10^6^	2.15 × 10^6^
F12	Std	1.90 × 10^5^	6.03 × 10^6^	1.36 × 10^9^	1.03 × 10^6^	2.59 × 10^7^	2.20 × 10^9^	1.00 × 10^8^	1.11 × 10^8^
F12	Avg	2.12 × 10^5^	7.12 × 10^6^	4.53 × 10^8^	1.29 × 10^6^	3.31 × 10^7^	6.43 × 10^9^	9.59 × 10^7^	1.10 × 10^8^
F12	Time	2.31 × 10^−2^	5.07 × 10^−2^	3.00 × 10^−2^	3.12 × 10^−2^	3.63 × 10^−2^	2.86 × 10^−2^	3.37 × 10^−2^	3.21 × 10^−1^
F13	Best	2.81 × 10^3^	1.63 × 10^4^	5.72 × 10^4^	4.17 × 10^3^	3.93 × 10^5^	6.95 × 10^7^	3.32 × 10^4^	2.80 × 10^4^
F13	Std	5.64 × 10^3^	5.16 × 10^4^	4.93 × 10^8^	1.53 × 10^4^	3.49 × 10^5^	1.41 × 10^9^	2.70 × 10^7^	1.72 × 10^5^
F13	Avg	8.85 × 10^3^	1.01 × 10^5^	9.74 × 10^7^	2.04 × 10^4^	7.30 × 10^5^	2.02 × 10^9^	9.70 × 10^6^	1.98 × 10^5^
F13	Time	1.68 × 10^−2^	4.05 × 10^−2^	2.53 × 10^−2^	2.48 × 10^−2^	2.80 × 10^−2^	1.99 × 10^−2^	2.78 × 10^−2^	3.39 × 10^−1^
F14	Best	1.50 × 10^3^	2.57 × 10^3^	1.73 × 10^3^	3.07 × 10^3^	1.08 × 10^4^	4.80 × 10^4^	1.91 × 10^3^	8.20 × 10^3^
F14	Std	3.91 × 10^2^	2.64 × 10^4^	8.06 × 10^3^	4.54 × 10^4^	5.28 × 10^5^	5.28 × 10^5^	6.01 × 10^5^	3.04 × 10^5^
F14	Avg	1.59 × 10^3^	3.57 × 10^4^	6.79 × 10^3^	5.88 × 10^4^	6.00 × 10^5^	6.86 × 10^5^	3.81 × 10^5^	2.31 × 10^5^
F14	Time	1.85 × 10^−2^	3.97 × 10^−2^	2.48 × 10^−2^	2.48 × 10^−2^	3.38 × 10^−2^	2.98 × 10^−2^	3.39 × 10^−2^	3.70 × 10^−1^
F15	Best	1.61 × 10^3^	1.27 × 10^4^	8.77 × 10^3^	1.63 × 10^3^	1.68 × 10^4^	2.61 × 10^5^	2.48 × 10^4^	1.04 × 10^4^
F15	Std	4.64 × 10^2^	5.30 × 10^4^	1.37 × 10^6^	1.14 × 10^4^	6.09 × 10^4^	3.36 × 10^6^	1.17 × 10^7^	4.46 × 10^4^
F15	Avg	2.04 × 10^3^	5.71 × 10^4^	3.82 × 10^5^	1.01 × 10^4^	9.13 × 10^4^	3.05 × 10^6^	3.24 × 10^6^	3.04 × 10^4^
F15	Time	1.59 × 10^−2^	3.81 × 10^−2^	2.06 × 10^−2^	2.17 × 10^−2^	2.74 × 10^−2^	1.96 × 10^−2^	2.76 × 10^−2^	3.34 × 10^−1^
F16	Best	2.01 × 10^3^	2.12 × 10^3^	2.33 × 10^3^	1.74 × 10^3^	2.61 × 10^3^	3.15 × 10^3^	2.31 × 10^3^	2.83 × 10^3^
F16	Std	2.56 × 10^2^	3.24 × 10^2^	4.38 × 10^2^	3.77 × 10^2^	4.24 × 10^2^	4.44 × 10^2^	2.65 × 10^2^	4.44 × 10^2^
F16	Avg	2.48 × 10^3^	2.70 × 10^3^	3.07 × 10^3^	2.84 × 10^3^	3.35 × 10^3^	3.99 × 10^3^	2.63 × 10^3^	3.47 × 10^3^
F16	Time	1.82 × 10^−2^	4.27 × 10^−2^	2.57 × 10^−2^	3.02 × 10^−2^	3.56 × 10^−2^	2.24 × 10^−2^	2.91 × 10^−2^	3.53 × 10^−1^
F17	Best	1.76 × 10^3^	1.80 × 10^3^	2.01 × 10^3^	1.86 × 10^3^	2.34 × 10^3^	2.01 × 10^3^	1.80 × 10^3^	2.21 × 10^3^
F17	Std	8.58 × 10^1^	1.75 × 10^2^	2.30 × 10^2^	2.79 × 10^2^	2.66 × 10^2^	3.18 × 10^2^	1.44 × 10^2^	2.09 × 10^2^
F17	Avg	1.88 × 10^3^	2.10 × 10^3^	2.38 × 10^3^	2.57 × 10^3^	2.73 × 10^3^	2.76 × 10^3^	2.05 × 10^3^	2.53 × 10^3^
F17	Time	3.01 × 10^−2^	6.23 × 10^−2^	5.19 × 10^−2^	4.15 × 10^−2^	6.42 × 10^−2^	3.44 × 10^−2^	4.24 × 10^−2^	3.93 × 10^−1^
F18	Best	9.86 × 10^3^	1.07 × 10^5^	1.66 × 10^4^	8.71 × 10^4^	8.91 × 10^4^	1.03 × 10^5^	2.17 × 10^5^	2.00 × 10^5^
F18	Std	2.34 × 10^4^	4.88 × 10^5^	1.12 × 10^6^	1.06 × 10^6^	3.03 × 10^6^	2.98 × 10^6^	1.44 × 10^6^	7.45 × 10^5^
F18	Avg	4.81 × 10^4^	5.82 × 10^5^	3.50 × 10^5^	1.02 × 10^6^	2.16 × 10^6^	4.01 × 10^6^	1.23 × 10^6^	9.72 × 10^5^
F18	Time	2.21 × 10^−2^	4.81 × 10^−2^	2.78 × 10^−2^	2.70 × 10^−2^	3.56 × 10^−2^	2.76 × 10^−2^	3.34 × 10^−2^	3.79 × 10^−1^
F19	Best	1.96 × 10^3^	7.28 × 10^3^	1.06 × 10^4^	1.95 × 10^3^	2.47 × 10^5^	1.07 × 10^7^	8.28 × 10^3^	4.43 × 10^5^
F19	Std	1.73 × 10^3^	3.64 × 10^5^	5.68 × 10^5^	1.18 × 10^4^	4.95 × 10^5^	2.15 × 10^8^	2.42 × 10^7^	9.78 × 10^5^
F19	Avg	2.70 × 10^3^	2.96 × 10^5^	2.64 × 10^5^	8.72 × 10^3^	8.67 × 10^5^	2.24 × 10^8^	5.47 × 10^6^	1.86 × 10^6^
F19	Time	5.18 × 10^−2^	8.92 × 10^−2^	9.13 × 10^−2^	7.31 × 10^−2^	1.08 × 10^−1^	5.48 × 10^−2^	6.26 × 10^−2^	4.67 × 10^−1^
F20	Best	2.06 × 10^3^	2.24 × 10^3^	2.30 × 10^3^	2.52 × 10^3^	2.39 × 10^3^	2.37 × 10^3^	2.19 × 10^3^	2.37 × 10^3^
F20	Std	8.81 × 10^1^	1.20 × 10^2^	2.13 × 10^2^	2.30 × 10^2^	1.91 × 10^2^	1.50 × 10^2^	1.19 × 10^2^	1.04 × 10^2^
F20	Avg	2.22 × 10^3^	2.42 × 10^3^	2.60 × 10^3^	2.85 × 10^3^	2.79 × 10^3^	2.67 × 10^3^	2.39 × 10^3^	2.58 × 10^3^
F20	Time	6.57 × 10^−2^	5.14 × 10^−2^	3.86 × 10^−2^	3.38 × 10^−2^	5.06 × 10^−2^	2.94 × 10^−2^	3.56 × 10^−2^	3.69 × 10^−1^
F21	Best	2.20 × 10^3^	2.20 × 10^3^	2.24 × 10^3^	2.20 × 10^3^	2.24 × 10^3^	2.82 × 10^3^	2.24 × 10^3^	2.28 × 10^3^
F21	Std	6.90 × 10^2^	6.52 × 10^2^	1.05 × 10^3^	7.86 × 10^2^	9.09 × 10^2^	3.81 × 10^2^	4.01 × 10^2^	9.74 × 10^2^
F21	Avg	2.61 × 10^3^	2.88 × 10^3^	3.72 × 10^3^	4.08 × 10^3^	3.90 × 10^3^	3.23 × 10^3^	3.17 × 10^3^	3.66 × 10^3^
F21	Time	3.24 × 10^−2^	6.49 × 10^−2^	5.61 × 10^−2^	4.42 × 10^−2^	6.17 × 10^−2^	3.88 × 10^−2^	4.67 × 10^−2^	3.87 × 10^−1^
F22	Best	1.44 × 10^4^	8.74 × 10^3^	1.08 × 10^4^	1.17 × 10^4^	1.27 × 10^4^	1.64 × 10^4^	7.25 × 10^3^	1.18 × 10^4^
F22	Std	1.19 × 10^3^	2.15 × 10^3^	3.11 × 10^3^	1.85 × 10^3^	1.48 × 10^3^	1.75 × 10^3^	3.04 × 10^3^	1.63 × 10^3^
F22	Avg	1.73 × 10^4^	1.19 × 10^4^	1.48 × 10^4^	1.50 × 10^4^	1.60 × 10^4^	2.03 × 10^4^	1.20 × 10^4^	1.51 × 10^4^
F22	Time	3.20 × 10^−2^	5.42 × 10^−2^	5.07 × 10^−2^	3.83 × 10^−2^	6.29 × 10^−2^	3.57 × 10^−2^	4.19 × 10^−2^	3.69 × 10^−1^
F23	Best	3.60 × 10^3^	2.40 × 10^3^	5.15 × 10^3^	4.37 × 10^3^	5.75 × 10^3^	5.68 × 10^3^	3.17 × 10^3^	4.45 × 10^3^
F23	Std	2.15 × 10^2^	4.03 × 10^2^	8.13 × 10^2^	7.16 × 10^2^	8.55 × 10^2^	3.99 × 10^2^	3.69 × 10^2^	6.52 × 10^2^
F23	Avg	4.29 × 10^3^	3.83 × 10^3^	6.71 × 10^3^	5.68 × 10^3^	7.20 × 10^3^	6.41 × 10^3^	3.90 × 10^3^	5.96 × 10^3^
F23	Time	3.21 × 10^−2^	5.50 × 10^−2^	5.41 × 10^−2^	4.19 × 10^−2^	6.70 × 10^−2^	3.70 × 10^−2^	4.35 × 10^−2^	3.99 × 10^−1^
F24	Best	2.50 × 10^3^	2.60 × 10^3^	2.89 × 10^3^	5.64 × 10^3^	5.21 × 10^3^	4.33 × 10^3^	3.02 × 10^3^	4.57 × 10^3^
F24	Std	9.91 × 10^2^	7.28 × 10^2^	6.56 × 10^2^	1.01 × 10^2^	2.07 × 10^2^	6.10 × 10^2^	6.29 × 10^2^	2.47 × 10^2^
F24	Avg	3.37 × 10^3^	3.45 × 10^3^	5.52 × 10^3^	5.84 × 10^3^	5.79 × 10^3^	5.90 × 10^3^	4.19 × 10^3^	5.72 × 10^3^
F24	Time	3.31 × 10^−2^	5.46 × 10^−2^	5.27 × 10^−2^	4.00 × 10^−2^	6.20 × 10^−2^	3.46 × 10^−2^	4.48 × 10^−2^	3.86 × 10^−1^
F25	Best	2.88 × 10^3^	2.88 × 10^3^	2.92 × 10^3^	2.88 × 10^3^	2.90 × 10^3^	3.70 × 10^3^	2.93 × 10^3^	2.92 × 10^3^
F25	Std	1.18 × 10^1^	1.38 × 10^1^	5.84 × 10^2^	1.15 × 10^1^	2.89 × 10^1^	3.13 × 10^2^	4.09 × 10^1^	3.38 × 10^1^
F25	Avg	2.89 × 10^3^	2.89 × 10^3^	3.28 × 10^3^	2.89 × 10^3^	2.95 × 10^3^	4.11 × 10^3^	3.00 × 10^3^	3.00 × 10^3^
F25	Time	3.43 × 10^−2^	5.43 × 10^−2^	5.05 × 10^−2^	4.13 × 10^−2^	6.45 × 10^−2^	3.45 × 10^−2^	4.37 × 10^−2^	3.71 × 10^−1^
F26	Best	2.80 × 10^3^	2.80 × 10^3^	3.46 × 10^3^	2.90 × 10^3^	3.46 × 10^3^	6.13 × 10^3^	3.58 × 10^3^	3.52 × 10^3^
F26	Std	1.07 × 10^3^	1.02 × 10^3^	2.07 × 10^3^	1.60 × 10^3^	1.53 × 10^3^	6.98 × 10^2^	5.29 × 10^2^	1.63 × 10^3^
F26	Avg	4.31 × 10^3^	4.14 × 10^3^	7.43 × 10^3^	6.52 × 10^3^	7.45 × 10^3^	7.52 × 10^3^	4.78 × 10^3^	6.93 × 10^3^
F26	Time	3.78 × 10^−2^	5.99 × 10^−2^	6.27 × 10^−2^	4.74 × 10^−2^	7.79 × 10^−2^	4.22 × 10^−2^	4.97 × 10^−2^	3.99 × 10^−1^
F27	Best	3.21 × 10^3^	3.21 × 10^3^	3.23 × 10^3^	3.22 × 10^3^	3.25 × 10^3^	3.36 × 10^3^	3.22 × 10^3^	3.28 × 10^3^
F27	Std	9.28 × 10^0^	2.23 × 10^1^	1.24 × 10^2^	5.27 × 10^1^	2.55 × 10^2^	1.37 × 10^2^	2.07 × 10^1^	9.35 × 10^1^
F27	Avg	3.23 × 10^3^	3.24 × 10^3^	3.38 × 10^3^	3.30 × 10^3^	3.56 × 10^3^	3.65 × 10^3^	3.26 × 10^3^	3.44 × 10^3^
F27	Time	3.91 × 10^−2^	6.25 × 10^−2^	7.15 × 10^−2^	5.26 × 10^−2^	9.13 × 10^−2^	4.80 × 10^−2^	5.36 × 10^−2^	4.17 × 10^−1^
F28	Best	3.20 × 10^3^	3.19 × 10^3^	3.25 × 10^3^	3.20 × 10^3^	3.26 × 10^3^	4.48 × 10^3^	3.29 × 10^3^	3.28 × 10^3^
F28	Std	1.86 × 10^1^	2.62 × 10^1^	7.76 × 10^2^	1.96 × 10^1^	2.48 × 10^1^	4.87 × 10^2^	1.01 × 10^2^	6.83 × 10^1^
F28	Avg	3.23 × 10^3^	3.24 × 10^3^	3.64 × 10^3^	3.22 × 10^3^	3.32 × 10^3^	5.23 × 10^3^	3.48 × 10^3^	3.38 × 10^3^
F28	Time	3.69 × 10^−2^	6.08 × 10^−2^	6.51 × 10^−2^	4.79 × 10^−2^	7.32 × 10^−2^	3.89 × 10^−2^	4.70 × 10^−2^	4.00 × 10^−1^
F29	Best	3.48 × 10^3^	3.53 × 10^3^	3.79 × 10^3^	3.72 × 10^3^	4.06 × 10^3^	4.11 × 10^3^	3.58 × 10^3^	3.92 × 10^3^
F29	Std	1.33 × 10^2^	2.25 × 10^2^	4.96 × 10^2^	3.16 × 10^2^	2.94 × 10^2^	3.51 × 10^2^	1.91 × 10^2^	4.84 × 10^2^
F29	Avg	3.71 × 10^3^	3.90 × 10^3^	4.55 × 10^3^	4.21 × 10^3^	4.63 × 10^3^	4.84 × 10^3^	3.89 × 10^3^	4.88 × 10^3^
F29	Time	3.56 × 10^−2^	6.07 × 10^−2^	6.05 × 10^−2^	4.66 × 10^−2^	8.28 × 10^−2^	5.16 × 10^−2^	5.36 × 10^−2^	4.37 × 10^−1^
F30	Best	8.98 × 10^3^	5.21 × 10^5^	2.60 × 10^5^	6.85 × 10^3^	5.74 × 10^5^	2.76 × 10^7^	8.89 × 10^5^	2.50 × 10^6^
F30	Std	5.87 × 10^3^	1.50 × 10^6^	9.09 × 10^7^	6.22 × 10^3^	3.81 × 10^6^	1.41 × 10^8^	1.09 × 10^7^	3.00 × 10^7^
F30	Avg	1.79 × 10^4^	2.66 × 10^6^	3.31 × 10^7^	1.39 × 10^4^	5.12 × 10^6^	1.26 × 10^8^	1.24 × 10^7^	2.25 × 10^7^
F30	Time	5.02 × 10^−2^	7.58 × 10^−2^	9.33 × 10^−2^	7.08 × 10^−2^	1.22 × 10^−1^	5.61 × 10^−2^	6.28 × 10^−2^	4.32 × 10^−1^

**Table 2:** Ranking matrix of 8 algorithms CEC2017.

Function	EAOO	AOO	BKA	SSA	HHO	DBO	GWO	HO
F1	2	3	7	1	4	8	6	5
F3	3	1	2	5	4	8	7	6
F4	2	3	7	1	4	8	5	6
F5	3	1	6	7	5	8	2	4
F6	1	3	6	4	7	8	2	5
F7	3	1	6	7	8	5	2	4
F8	3	2	5	7	6	8	1	4
F9	1	3	4	5	8	7	2	6
F10	7	1	5	4	6	8	2	3
F11	2	1	4	3	5	8	7	6
F12	1	3	7	2	4	8	5	6
F13	1	3	7	2	5	8	6	4
F14	1	3	2	4	7	8	6	5
F15	1	4	6	2	5	7	8	3
F16	1	3	5	4	6	8	2	7
F17	1	3	4	6	7	8	2	5
F18	1	3	2	5	7	8	6	4
F19	1	4	3	2	5	8	7	6
F20	1	3	5	8	7	6	2	4
F21	1	2	6	8	7	4	3	5
F22	7	1	3	4	6	8	2	5
F23	3	1	7	4	8	6	2	5
F24	1	2	4	7	6	8	3	5
F25	3	2	7	1	4	8	5	6
F26	2	1	6	4	7	8	3	5
F27	1	2	5	4	7	8	3	6
F28	2	3	7	1	4	8	6	5
F29	1	3	5	4	6	7	2	8
F30	2	3	7	1	4	8	5	6

**Table 3:** Comparison of Wilcoxon rank sum results for CEC2017.

Function	AOO	BKA	SSA	HHO	DBO	GWO	HO
F1	3.59 × 10^−5^	3.02 × 10^−11^	8.30 × 10^−1^	3.02 × 10^−11^	3.02 × 10^−11^	3.02 × 10^−11^	3.02 × 10^−11^
F3	3.02 × 10^−11^	8.84 × 10^−7^	6.35 × 10^−2^	1.30 × 10^−1^	8.15 × 10^−11^	2.88 × 10^−6^	1.64 × 10^−5^
F4	3.33 × 10^−1^	1.61 × 10^−10^	2.28 × 10^−1^	1.70 × 10^−8^	3.02 × 10^−11^	3.20 × 10^−9^	3.02 × 10^−11^
F5	6.20 × 10^−4^	3.82 × 10^−10^	3.02 × 10^−11^	4.50 × 10^−11^	3.02 × 10^−11^	2.43 × 10^−5^	7.39 × 10^−11^
F6	3.02 × 10^−11^	3.02 × 10^−11^	3.02 × 10^−11^	3.02 × 10^−11^	3.02 × 10^−11^	3.02 × 10^−11^	3.02 × 10^−11^
F7	5.46 × 10^−6^	3.02 × 10^−11^	3.02 × 10^−11^	3.02 × 10^−11^	3.02 × 10^−11^	6.55 × 10^−4^	3.02 × 10^−11^
F8	9.83 × 10^−8^	8.12 × 10^−4^	5.09 × 10^−6^	4.94 × 10^−5^	3.02 × 10^−11^	1.31 × 10^−8^	3.03 × 10^−3^
F9	1.61 × 10^−10^	3.02 × 10^−11^	3.02 × 10^−11^	3.02 × 10^−11^	3.02 × 10^−11^	3.82 × 10^−10^	3.02 × 10^−11^
F10	3.69 × 10^−11^	3.82 × 10^−9^	1.78 × 10^−10^	3.35 × 10^−8^	1.96 × 10^−10^	1.07 × 10^−7^	4.50 × 10^−11^
F11	3.37 × 10^−5^	3.95 × 10^−1^	8.07 × 10^−1^	2.43 × 10^−5^	3.02 × 10^−11^	3.02 × 10^−11^	3.02 × 10^−11^
F12	7.39 × 10^−11^	3.02 × 10^−11^	2.78 × 10^−7^	3.02 × 10^−11^	3.02 × 10^−11^	3.02 × 10^−11^	3.02 × 10^−11^
F13	4.08 × 10^−11^	3.02 × 10^−11^	4.46 × 10^−4^	3.02 × 10^−11^	3.02 × 10^−11^	3.02 × 10^−11^	3.34 × 10^−11^
F14	3.34 × 10^−11^	1.46 × 10^−10^	3.34 × 10^−11^	3.02 × 10^−11^	3.02 × 10^−11^	3.34 × 10^−11^	3.02 × 10^−11^
F15	3.02 × 10^−11^	3.02 × 10^−11^	4.80 × 10^−7^	3.02 × 10^−11^	3.02 × 10^−11^	3.02 × 10^−11^	3.02 × 10^−11^
F16	2.42 × 10^−2^	1.07 × 10^−7^	6.77 × 10^−5^	7.38 × 10^−10^	3.02 × 10^−11^	1.45 × 10^−1^	3.69 × 10^−11^
F17	1.61 × 10^−6^	4.98 × 10^−11^	3.16 × 10^−10^	3.02 × 10^−11^	4.08 × 10^−11^	2.32 × 10^−6^	3.02 × 10^−11^
F18	3.02 × 10^−11^	3.01 × 10^−4^	4.50 × 10^−11^	3.34 × 10^−11^	3.02 × 10^−11^	3.02 × 10^−11^	3.02 × 10^−11^
F19	3.34 × 10^−11^	3.69 × 10^−11^	5.46 × 10^−6^	3.02 × 10^−11^	3.02 × 10^−11^	3.34 × 10^−11^	3.02 × 10^−11^
F20	1.56 × 10^−8^	1.61 × 10^−10^	3.02 × 10^−11^	3.69 × 10^−11^	5.49 × 10^−11^	3.01 × 10^−7^	7.39 × 10^−11^
F21	7.73 × 10^−1^	1.25 × 10^−7^	1.07 × 10^−7^	5.97 × 10^−9^	5.87 × 10^−4^	1.24 × 10^−3^	9.06 × 10^−8^
F22	6.12 × 10^−10^	3.59 × 10^−5^	1.73 × 10^−6^	6.91 × 10^−4^	1.25 × 10^−7^	9.26 × 10^−9^	1.39 × 10^−6^
F23	8.20 × 10^−7^	3.02 × 10^−11^	1.61 × 10^−10^	3.02 × 10^−11^	3.02 × 10^−11^	7.74 × 10^−6^	5.49 × 10^−11^
F24	1.67 × 10^−1^	2.15 × 10^−10^	3.02 × 10^−11^	3.02 × 10^−11^	1.96 × 10^−10^	7.29 × 10^−3^	4.98 × 10^−11^
F25	4.04 × 10^−1^	3.69 × 10^−11^	1.81 × 10^−1^	8.15 × 10^−11^	3.02 × 10^−11^	3.02 × 10^−11^	3.34 × 10^−11^
F26	3.04 × 10^−1^	3.09 × 10^−6^	9.83 × 10^−8^	8.48 × 10^−9^	3.02 × 10^−11^	8.53 × 10^−1^	1.11 × 10^−6^
F27	3.95 × 10^−1^	3.47 × 10^−10^	1.56 × 10^−8^	3.02 × 10^−11^	3.02 × 10^−11^	5.86 × 10^−6^	3.02 × 10^−11^
F28	4.21 × 10^−2^	6.07 × 10^−11^	8.50 × 10^−2^	3.69 × 10^−11^	3.02 × 10^−11^	3.02 × 10^−11^	3.02 × 10^−11^
F29	5.56 × 10^−4^	7.39 × 10^−11^	1.29 × 10^−9^	3.02 × 10^−11^	3.02 × 10^−11^	2.53 × 10^−4^	3.69 × 10^−11^
F30	3.02 × 10^−11^	3.02 × 10^−11^	3.03 × 10^−3^	3.02 × 10^−11^	3.02 × 10^−11^	3.02 × 10^−11^	3.02 × 10^−11^

**Table 4:** Win_Tie_Loss statistics for CEC2017.

Function	AOO	BKA	SSA	HHO	DBO	GWO	HO
F1	+	+	=	+	+	+	+
F3	−	−	=	=	+	+	+
F4	=	+	=	+	+	+	+
F5	−	+	+	+	+	−	+
F6	+	+	+	+	+	+	+
F7	−	+	+	+	+	−	+
F8	−	+	+	+	+	−	+
F9	+	+	+	+	+	+	+
F10	−	−	−	−	+	−	−
F11	−	=	=	+	+	+	+
F12	+	+	+	+	+	+	+
F13	+	+	+	+	+	+	+
F14	+	+	+	+	+	+	+
F15	+	+	+	+	+	+	+
F16	+	+	+	+	+	=	+
F17	+	+	+	+	+	+	+
F18	+	+	+	+	+	+	+
F19	+	+	+	+	+	+	+
F20	+	+	+	+	+	+	+
F21	=	+	+	+	+	+	+
F22	−	−	−	−	+	−	−
F23	−	+	+	+	+	−	+
F24	=	+	+	+	+	+	+
F25	=	+	=	+	+	+	+
F26	=	+	+	+	+	=	+
F27	=	+	+	+	+	+	+
F28	+	+	=	+	+	+	+
F29	+	+	+	+	+	+	+
F30	+	+	−	+	+	+	+

**Table 5:** Results of different swarm intelligence algorithms of CEC 2020.

Function	Metric	EAOO	AOO	BKA	SSA	HHO	DBO	GWO	HO
F1	Best	1.06 × 10^2^	2.14 × 10^3^	9.85 × 10^7^	1.08 × 10^2^	1.43 × 10^7^	2.34 × 10^10^	4.67 × 10^8^	8.38 × 10^5^
F1	Std	2.91 × 10^3^	4.43 × 10^3^	1.67 × 10^10^	5.58 × 10^3^	8.66 × 10^6^	2.97 × 10^9^	1.54 × 10^9^	2.08 × 10^8^
F1	Avg	3.15 × 10^3^	6.13 × 10^3^	1.16 × 10^10^	4.53 × 10^3^	3.09 × 10^7^	2.82 × 10^10^	2.84 × 10^9^	1.95 × 10^8^
F1	Time	2.83 × 10^−2^	4.68 × 10^−2^	2.85 × 10^−2^	3.30 × 10^−2^	4.00 × 10^−2^	3.00 × 10^−2^	2.94 × 10^−2^	3.36 × 10^−1^
F2	Best	5.94 × 10^3^	3.38 × 10^3^	3.81 × 10^3^	4.52 × 10^3^	4.38 × 10^3^	6.62 × 10^3^	3.76 × 10^3^	3.81 × 10^3^
F2	Std	3.34 × 10^2^	6.57 × 10^2^	1.02 × 10^3^	7.25 × 10^2^	6.59 × 10^2^	7.52 × 10^2^	6.07 × 10^2^	4.59 × 10^2^
F2	Avg	6.79 × 10^3^	4.66 × 10^3^	5.50 × 10^3^	5.57 × 10^3^	5.62 × 10^3^	8.05 × 10^3^	4.62 × 10^3^	4.95 × 10^3^
F2	Time	2.19 × 10^−2^	4.23 × 10^−2^	3.09 × 10^−2^	2.64 × 10^−2^	3.94 × 10^−2^	2.41 × 10^−2^	3.12 × 10^−2^	3.11 × 10^−1^
F3	Best	8.58 × 10^2^	7.95 × 10^2^	1.07 × 10^3^	1.02 × 10^3^	1.12 × 10^3^	1.15 × 10^3^	8.08 × 10^2^	9.69 × 10^2^
F3	Std	1.85 × 10^1^	2.91 × 10^1^	8.15 × 10^1^	9.05 × 10^1^	7.41 × 10^1^	4.80 × 10^1^	6.64 × 10^1^	7.04 × 10^1^
F3	Avg	8.99 × 10^2^	8.57 × 10^2^	1.21 × 10^3^	1.24 × 10^3^	1.26 × 10^3^	1.22 × 10^3^	9.04 × 10^2^	1.14 × 10^3^
F3	Time	1.82 × 10^−2^	3.98 × 10^−2^	2.61 × 10^−2^	2.26 × 10^−2^	3.23 × 10^−2^	1.91 × 10^−2^	2.82 × 10^−2^	3.03 × 10^−1^
F4	Best	1.91 × 10^3^	1.91 × 10^3^	1.94 × 10^3^	1.92 × 10^3^	1.93 × 10^3^	6.87 × 10^4^	1.91 × 10^3^	2.05 × 10^3^
F4	Std	2.13 × 10^0^	2.80 × 10^0^	4.82 × 10^5^	1.03 × 10^1^	1.19 × 10^1^	6.83 × 10^5^	3.03 × 10^3^	3.81 × 10^2^
F4	Avg	1.92 × 10^3^	1.91 × 10^3^	1.56 × 10^5^	1.94 × 10^3^	1.95 × 10^3^	1.03 × 10^6^	3.70 × 10^3^	2.49 × 10^3^
F4	Time	1.73 × 10^−2^	3.76 × 10^−2^	2.56 × 10^−2^	2.31 × 10^−2^	3.12 × 10^−2^	2.04 × 10^−2^	2.88 × 10^−2^	3.08 × 10^−1^
F5	Best	2.90 × 10^4^	9.87 × 10^4^	3.47 × 10^4^	1.28 × 10^5^	3.33 × 10^5^	3.98 × 10^6^	4.35 × 10^5^	8.04 × 10^5^
F5	Std	2.40 × 10^5^	5.01 × 10^5^	5.60 × 10^6^	5.99 × 10^5^	4.42 × 10^6^	1.21 × 10^7^	2.93 × 10^6^	3.56 × 10^6^
F5	Avg	2.50 × 10^5^	7.00 × 10^5^	2.24 × 10^6^	8.48 × 10^5^	5.00 × 10^6^	2.25 × 10^7^	3.49 × 10^6^	5.71 × 10^6^
F5	Time	1.98 × 10^−2^	3.90 × 10^−2^	2.79 × 10^−2^	2.54 × 10^−2^	3.47 × 10^−2^	2.19 × 10^−2^	2.89 × 10^−2^	2.94 × 10^−1^
F6	Best	2.02 × 10^3^	1.77 × 10^3^	1.94 × 10^3^	1.93 × 10^3^	2.06 × 10^3^	2.67 × 10^3^	1.78 × 10^3^	2.08 × 10^3^
F6	Std	1.64 × 10^2^	2.57 × 10^2^	7.49 × 10^2^	3.22 × 10^2^	6.61 × 10^2^	3.72 × 10^2^	2.63 × 10^2^	3.77 × 10^2^
F6	Avg	2.28 × 10^3^	2.17 × 10^3^	2.92 × 10^3^	2.54 × 10^3^	2.91 × 10^3^	3.38 × 10^3^	2.11 × 10^3^	2.94 × 10^3^
F6	Time	1.90 × 10^−2^	3.96 × 10^−2^	2.65 × 10^−2^	2.42 × 10^−2^	3.34 × 10^−2^	2.05 × 10^−2^	2.95 × 10^−2^	3.19 × 10^−1^
F7	Best	3.74 × 10^3^	1.54 × 10^4^	1.46 × 10^4^	3.85 × 10^4^	7.44 × 10^4^	7.26 × 10^5^	1.16 × 10^5^	7.73 × 10^4^
F7	Std	1.85 × 10^4^	1.21 × 10^5^	2.57 × 10^5^	1.69 × 10^5^	7.68 × 10^5^	3.60 × 10^6^	2.00 × 10^6^	1.46 × 10^6^
F7	Avg	1.79 × 10^4^	1.62 × 10^5^	1.66 × 10^5^	3.05 × 10^5^	8.88 × 10^5^	5.46 × 10^6^	1.18 × 10^6^	1.58 × 10^6^
F7	Time	1.89 × 10^−2^	3.93 × 10^−2^	2.74 × 10^−2^	2.62 × 10^−2^	3.46 × 10^−2^	2.16 × 10^−2^	2.91 × 10^−2^	3.22 × 10^−1^
F8	Best	2.30 × 10^3^	2.30 × 10^3^	2.53 × 10^3^	2.30 × 10^3^	2.33 × 10^3^	5.05 × 10^3^	2.47 × 10^3^	2.43 × 10^3^
F8	Std	4.46 × 10^−1^	2.03 × 10^3^	1.73 × 10^3^	1.98 × 10^3^	1.44 × 10^3^	4.80 × 10^2^	2.12 × 10^3^	2.09 × 10^3^
F8	Avg	2.30 × 10^3^	3.88 × 10^3^	6.20 × 10^3^	6.45 × 10^3^	7.07 × 10^3^	5.95 × 10^3^	4.79 × 10^3^	5.15 × 10^3^
F8	Time	3.07 × 10^−2^	5.21 × 10^−2^	5.21 × 10^−2^	4.01 × 10^−2^	6.51 × 10^−2^	3.51 × 10^−2^	4.18 × 10^−2^	3.67 × 10^−1^
F9	Best	2.88 × 10^3^	2.89 × 10^3^	3.01 × 10^3^	2.92 × 10^3^	3.18 × 10^3^	3.15 × 10^3^	2.87 × 10^3^	3.05 × 10^3^
F9	Std	3.07 × 10^1^	4.24 × 10^1^	1.12 × 10^2^	9.82 × 10^1^	1.41 × 10^2^	9.23 × 10^1^	6.13 × 10^1^	9.55 × 10^1^
F9	Avg	2.96 × 10^3^	2.94 × 10^3^	3.24 × 10^3^	3.15 × 10^3^	3.45 × 10^3^	3.33 × 10^3^	2.96 × 10^3^	3.21 × 10^3^
F9	Time	3.35 × 10^−2^	5.44 × 10^−2^	5.58 × 10^−2^	4.23 × 10^−2^	6.95 × 10^−2^	3.71 × 10^−2^	4.43 × 10^−2^	3.60 × 10^−1^
F10	Best	2.88 × 10^3^	2.88 × 10^3^	2.93 × 10^3^	2.88 × 10^3^	2.90 × 10^3^	3.62 × 10^3^	2.95 × 10^3^	2.97 × 10^3^
F10	Std	1.21 × 10^1^	1.82 × 10^1^	1.69 × 10^2^	1.62 × 10^1^	2.58 × 10^1^	2.72 × 10^2^	4.71 × 10^1^	3.16 × 10^1^
F10	Avg	2.90 × 10^3^	2.90 × 10^3^	3.02 × 10^3^	2.90 × 10^3^	2.94 × 10^3^	4.04 × 10^3^	3.01 × 10^3^	3.02 × 10^3^
F10	Time	3.21 × 10^−2^	5.48 × 10^−2^	5.58 × 10^−2^	4.21 × 10^−2^	6.72 × 10^−2^	3.63 × 10^−2^	4.32 × 10^−2^	3.58 × 10^−1^

**Table 6:** Ranking matrix of 8 algorithms CEC2020.

Function	EAOO	AOO	BKA	SSA	HHO	DBO	GWO	HO
F1	1	3	7	2	4	8	6	5
F2	6	2	4	5	7	8	1	3
F3	2	1	5	7	8	6	3	4
F4	2	1	7	3	4	8	6	5
F5	1	2	4	3	6	8	5	7
F6	3	2	6	4	5	8	1	7
F7	1	2	3	4	5	8	6	7
F8	1	2	6	7	8	5	3	4
F9	3	1	6	4	8	7	2	5
F10	1	3	7	2	4	8	5	6

**Table 7:** Comparison of Wilcoxon rank sum results for CEC2020.

Function	AOO	BKA	SSA	HHO	DBO	GWO	HO
F1	2.16 × 10^−3^	3.02 × 10^−11^	8.42 × 10^−1^	3.02 × 10^−11^	3.02 × 10^−11^	3.02 × 10^−11^	3.0 × 10^−11^
F2	4.08 × 10^−11^	2.15 × 10^−6^	7.77 × 10^−9^	2.67 × 10^−9^	8.48 × 10^−9^	4.50 × 10^−11^	3.02 × 10^−11^
F3	2.20 × 10^−7^	3.02 × 10^−11^	3.02 × 10^−11^	3.01 × 10^−11^	3.02 × 10^−11^	6.52 × 10^−1^	3.01 × 10^−11^
F4	1.21 × 10^−10^	3.01 × 10^−11^	4.08 × 10^−11^	3.02 × 10^−11^	3.01 × 10^−11^	2.23 × 10^−9^	3.03 × 10^−11^
F5	4.64 × 10^−5^	1.22 × 10^−1^	1.86 × 10^−6^	6.70 × 10^−11^	3.02 × 10^−11^	6.07 × 10^−11^	4.50 × 10^−11^
F6	8.68 × 10^−3^	1.49 × 10^−6^	6.20 × 10^−4^	9.06 × 10^−8^	3.02 × 10^−11^	1.11 × 10^−3^	3.50 × 10^−9^
F7	9.76 × 10^−10^	1.85 × 10^−8^	4.08 × 10^−11^	3.69 × 10^−11^	3.01 × 10^−11^	3.02 × 10^−11^	3.34 × 10^−11^
F8	1.33 × 10^−10^	3.02 × 10^−11^	2.03 × 10^−9^	3.02 × 10^−11^	3.01 × 10^−11^	3.02 × 10^−11^	3.01 × 10^−11^
F9	4.23 × 10^−3^	3.02 × 10^−11^	3.82 × 10^−10^	3.02 × 10^−11^	3.03 × 10^−11^	1.54 × 10^−1^	3.01 × 10^−11^
F10	6.41 × 10^−1^	3.34 × 10^−11^	5.49 × 10^−1^	5.07 × 10^−10^	3.02 × 10^−11^	3.02 × 10^−11^	3.03 × 10^−11^

**Table 8:** Win_Tie_Loss Statistics of CEC2020.

Function	AOO	BKA	SSA	HHO	DBO	GWO	HO
F1	+	+	=	+	+	+	+
F2	−	−	−	−	+	−	−
F3	−	+	+	+	+	=	+
F4	−	+	+	+	+	+	+
F5	+	=	+	+	+	+	+
F6	−	+	+	+	+	−	+
F7	+	+	+	+	+	+	+
F8	+	+	+	+	+	+	+
F9	−	+	+	+	+	=	+
F30	=	+	=	+	+	+	+

**Table 9:** Comparison table of optimization results for welding beam design.

Welded Beam	EAOO	GWO	AOO	HO	BKA	SSA	HHO	DBO
Best	1.670 × 10^0^	1.672 × 10^0^	1.683 × 10^0^	−1.765 × 10^2^	1.671 × 10^0^	1.670 × 10^0^	1.733 × 10^0^	1.680 × 10^0^
Worst	1.674 × 10^0^	1.690 × 10^0^	1.750 × 10^0^	2.157 × 10^0^	2.304 × 10^0^	2.136 × 10^0^	2.437 × 10^0^	1.916 × 10^0^
Std	1.211 × 10^−3^	6.240 × 10^−3^	2.129 × 10^−2^	5.610 × 10^1^	1.984 × 10^−1^	1.480 × 10^−1^	2.026 × 10^−1^	7.285 × 10^−2^
Mean	1.671 × 10^0^	1.678 × 10^0^	1.702 × 10^0^	−3.476 × 10^1^	1.740 × 10^0^	1.762 × 10^0^	1.889 × 10^0^	1.797 × 10^0^
Median	1.670 × 10^0^	1.676 × 10^0^	1.692 × 10^0^	−1.108 × 10^1^	1.673 × 10^0^	1.675 × 10^0^	1.832 × 10^0^	1.776 × 10^0^
Time	1.415 × 10^−1^	1.196 × 10^−1^	1.767 × 10^−1^	8.498 × 10^−1^	2.579 × 10^−1^	1.755 × 10^−1^	2.596 × 10^−1^	1.487 × 10^−1^

**Table 10:** Comparison table of optimization results for pressure vessel design.

Pressure Vessel	EAOO	GWO	AOO	HO	BKA	SSA	HHO	DBO
Best	6.060 × 10^3^	6.063 × 10^3^	6.062 × 10^3^	6.489 × 10^3^	6.060 × 10^3^	6.060 × 10^3^	6.412 × 10^3^	6.108 × 10^3^
Worst	6.371 × 10^3^	7.277 × 10^3^	6.855 × 10^3^	1.106 × 10^4^	7.831 × 10^3^	7.337 × 10^3^	7.352 × 10^3^	7.566 × 10^3^
Std	9.641 × 10^1^	4.174 × 10^2^	3.570 × 10^2^	1.340 × 10^3^	5.825 × 10^2^	4.723 × 10^2^	2.917 × 10^2^	5.363 × 10^2^
Mean	6.099 × 10^3^	6.299 × 10^3^	6.333 × 10^3^	7.593 × 10^3^	6.706 × 10^3^	6.561 × 10^3^	6.871 × 10^3^	6.667 × 10^3^
Median	6.060 × 10^3^	6.080 × 10^3^	6.097 × 10^3^	7.270 × 10^3^	6.617 × 10^3^	6.398 × 10^3^	6.829 × 10^3^	6.520 × 10^3^
Time	1.164 × 10^−1^	1.090 × 10^−1^	1.470 × 10^−1^	8.356 × 10^−1^	1.990 × 10^−1^	1.547 × 10^−1^	2.429 × 10^−1^	1.361 × 10^−1^

## Data Availability

The data that support the findings of this study are available from the corresponding author upon request.
